# Precisely timed regulation of enhancer activity defines the binary expression pattern of Fushi tarazu in the *Drosophila* embryo

**DOI:** 10.1016/j.cub.2023.04.005

**Published:** 2023-07-24

**Authors:** Anthony Birnie, Audrey Plat, Cemil Korkmaz, Jacques P. Bothma

**Affiliations:** 1 Hubrecht Institute-KNAW, Uppsalalaan 8, 3584 CT Utrecht, the Netherlands

**Keywords:** transcription regulation, pattern formation, cell fate decisions, enhancers, timing, live imaging, quantitative biology, development, pioneer factors, chromatin biology

## Abstract

The genes that drive development each typically have many different enhancers. Properly coordinating the action of these different enhancers is crucial to correctly specifying cell-fate decisions, yet it remains poorly understood how their activity is choregraphed in time. To shed light on this question, we used recently developed single-cell live imaging tools to dissect the regulation of Fushi tarazu (Ftz) in *Drosophila melanogaster* embryos. Ftz is a transcription factor that is expressed in asymmetric stripes by two distinct enhancers: autoregulatory and zebra. The anterior edge of each stripe needs to be sharply defined to specify essential lineage boundaries. Here, we tracked how boundary cells commit to either a high-Ftz or low-Ftz fate by measuring Ftz protein traces in real time and simultaneously quantifying transcription from the endogenous locus and individual enhancers. This revealed that the autoregulatory enhancer does not establish this fate choice. Instead, it perpetuates the decision defined by zebra. This is contrary to the prevailing view that autoregulation drives the fate decision by causing bi-stable Ftz expression. Furthermore, we showed that the autoregulatory enhancer is not activated based on a Ftz-concentration threshold but through a timing-based mechanism. We hypothesize that this is regulated by several ubiquitously expressed pioneer-like transcription factors, which have recently been shown to act as timers in the embryo. Our work provides new insight into how precisely timed enhancer activity can directly regulate the dynamics of gene regulatory networks, which may be a general mechanism for ensuring that embryogenesis runs like clockwork.

## Highlights

• Ftz pattern formation occurs in two distinct transcriptional modes
• Ftz transcription transitions from short, dim bursts into longer, brighter bursts
• The two transcription modes are associated with activity of two distinct enhancers
• Ftz-dependent enhancer activity is time controlled in two separate examples

## Graphical abstract

**Figure:**
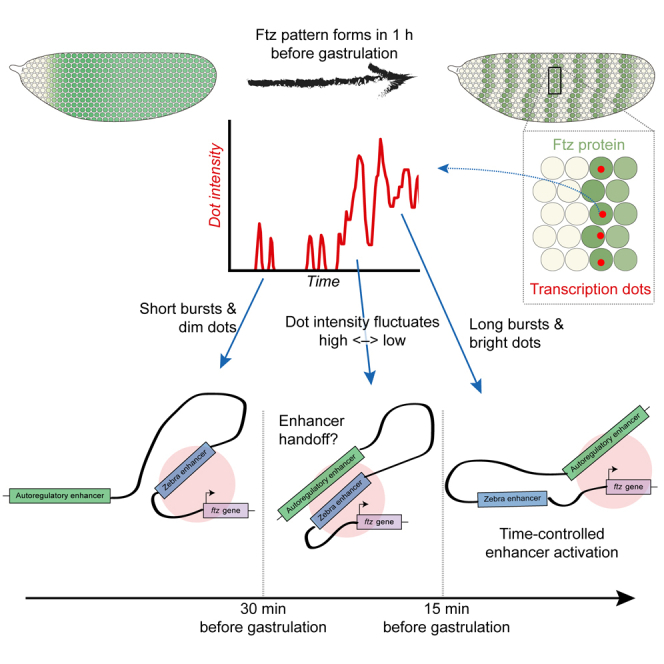

## Teaser

The coordination of enhancer activity is crucial for cell-fate decisions. Birnie and Plat et al. show in fly embryos that transcription of Ftz occurs in two distinct modes, matching the activity of its two enhancers. Also, activation of an Ftz-dependent enhancer is shown to be time controlled, possibly via pioneer-like factors opening up the chromatin.

## Introduction

**Figure 1:**
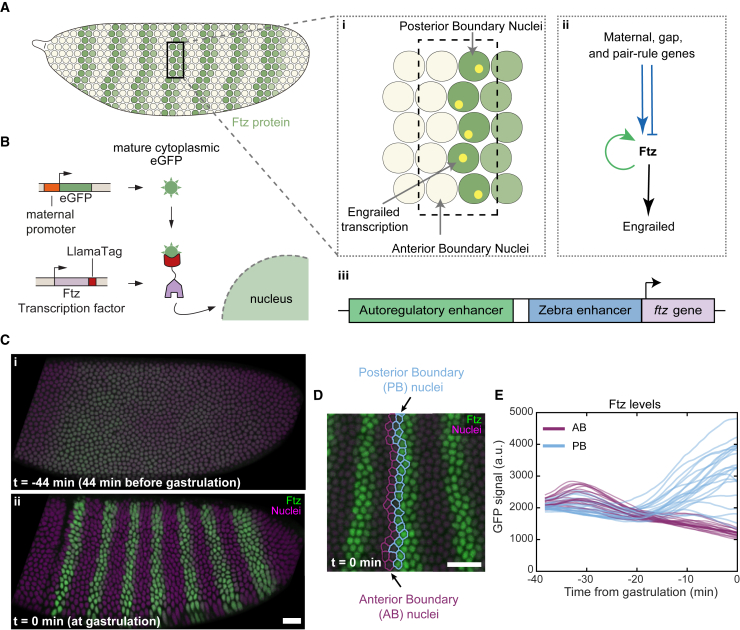
Visualizing Ftz protein dynamics in real time using the LlamaTag (A) Ftz expression in the fly embryo. (i, top) Anterior edge of each stripe shows binary expression with high Ftz posterior boundary (PB) nuclei and low Ftz anterior boundary (AB) nuclei. The Ftz target *engrailed* is expressed in the PB nuclei. (ii) Schematic of *ftz* regulatory network. (iii) *ftz* gene and its main enhancers. (B) The endogenous *ftz* gene is tagged with the LlamaTag. Maternally deposited cytoplasmic GFP binds the LlamaTagged Ftz protein and is then transported to the nucleus. (C) Ftz pattern dynamics. (D) Stripes 3, 4, and 5 at gastrulation. PB (AB) nuclei are outlined in blue (purple). (E) Nuclear GFP signal before background subtraction in boundary nuclei of stripe 4 of example embryo. Scale bars, 25 μm. See also Figures S1 and S6 and Video S1.

As genes direct cellular differentiation in animals, they often need to change which biological pathways they respond to.^1^ This is governed by the many different regulatory regions of DNA, called enhancers, which typically control the expression of an individual gene.^2^^,^^3^ How the action of multiple enhancers on a single gene is choregraphed in time remains poorly understood, mainly due to technical challenges in visualizing enhancer activity in real time. To understand how this enhancer coordination results in cell-fate specification *in vivo*, we examined the regulation of Fushi tarazu (Ftz), an important transcription factor that defines cell identity in the early fly embryo (Figure 1A).^4^^,^^5^^,^^6^ Initially, Ftz is expressed broadly, but over the course of an hour the pattern evolves into a series of asymmetric stripes.^7^^,^^8^ The anterior edge of each stripe consists of a single row of nuclei with high Ftz levels right next to a row of low-Ftz-level nuclei.^9^ This binary protein pattern specifies a compartment boundary that forms an essential foundation of the body plan.^6^^,^^10^^,^^11^ We refer to the high-Ftz row of cells as the posterior boundary (PB) nuclei because they are posterior of the future compartment boundary and, similarly, the row of neighboring low-Ftz nuclei are referred to as the anterior boundary (AB) nuclei (Figure 1Ai).

Ftz expression is defined by two distinct enhancers, autoregulatory and zebra,^4^^,^^5^^,^^12^^,^^13^^,^^14^ both of which are known to drive expression in all seven Ftz stripes^5^^,^^12^^,^^13^^,^^15^ (Figures 1Aiii and S1). Although each enhancer’s logic has been extensively characterized, there are two key open questions about how the activity of these enhancers is coordinated to specify the binary Ftz expression in the AB and PB nuclei. What role does each enhancer play in defining binary Ftz expression? The prevailing view is that zebra is activated first to produce graded bands of Ftz protein, which are then thresholded by the autoregulatory enhancer to produce binary Ftz expression.^5^^,^^13^^,^^14^^,^^16^ However, previous observations can also be explained by an opposing model where zebra defines the sharp expression pattern and the autoregulatory enhancer is activated later to simply maintain it. In short, the exact timing of the enhancer’s activation and repression in the boundary nuclei remains unknown.

The second pressing question is: how exactly is the autoregulatory enhancer regulated? Is it switched on passively by the zebra enhancer via the Ftz protein it produces, or is the timing of its initiation more directly regulated by another factor? Both enhancers are activated by various maternal products and repressed by the gap and pair-rule genes^5^^,^^12^^,^^13^^,^^15^^,^^16^^,^^17^^,^^18^ (Figure 1Aii). The autoregulatory enhancer also contains Ftz binding sites that are necessary for enhancer activation.^5^^,^^13^^,^^15^ Indeed, ubiquitous overexpression of Ftz protein leads to ectopic activation of *ftz* transcription, expanding the stripes.^19^^,^^20^ However, ectopic Ftz is not able to induce the autoregulatory enhancer in all embryonic nuclei because it is repressed by different pair-rule genes in the core of the inter-stripe regions.^16^ Ftz is thus clearly necessary for this enhancer to activate.^13^ However, as the autoregulatory enhancer is also bound by many other factors, if any of these are required for activation the enhancer would only become active after they bind.

## Results

### Boundary cells are identified at either side of the sharp anterior edges of Ftz stripes

To characterize Ftz dynamics, we fluorescently labeled the endogenous gene using CRISPR and the recently developed LlamaTag system^21^ (Figure 1B). Here, fluorescent GFP is ubiquitously present in the cytoplasm and, once a tagged transcription factor molecule is synthesized, it rapidly binds GFP. The binding specifically increases the fluorescence of GFP and causes it to be selectively translocated to the nucleus.^21^^,^^22^ This makes it possible to follow the Ftz pattern in living embryos using fluorescence confocal microcopy and accurately quantify transcription factor dynamics in real time,^21^ even for short-lived proteins like Ftz.^21^^,^^23^ The pattern started broad and then evolved into seven distinct stripes, consistent with previous observations^21^^,^^24^ (Figure 1C; Video S1). Time was measured relative to the onset of gastrulation in each embryo (Figure S1).

Video S1. Zoom-in video of Fushi tarazu protein and MCP-mCherry-NLS, related to Figure 1 Green is Ftz and magenta is nuclear mCherry signal. Scale bar is 20 μm.

To unambiguously identify boundary nuclei, we examined the refined pattern just before gastrulation. Nuclei were classified, based on their Ftz levels, into either a high-Ftz PB or a low-Ftz AB category (Figures 1D and S6). We then traced these nuclei back in time to see how they arrived at these different final states. In stripe 4, for example, the AB and PB nuclei are clearly different at gastrulation, but about 40 min earlier they are indistinguishable based on Ftz levels alone (Figure 1E). Although the Ftz levels of PB nuclei start to exceed AB nuclei levels around −20 min, it only becomes clear around −10 min which nuclei will become AB or PB, based on differing levels of Ftz. To understand how this is regulated we measured the transcriptional dynamics of *ftz* as nuclei made their fate choice.

### Dynamics of endogenous *ftz* transcription reveal that Ftz protein does not define the fate decision of boundary nuclei

**Figure 2:**
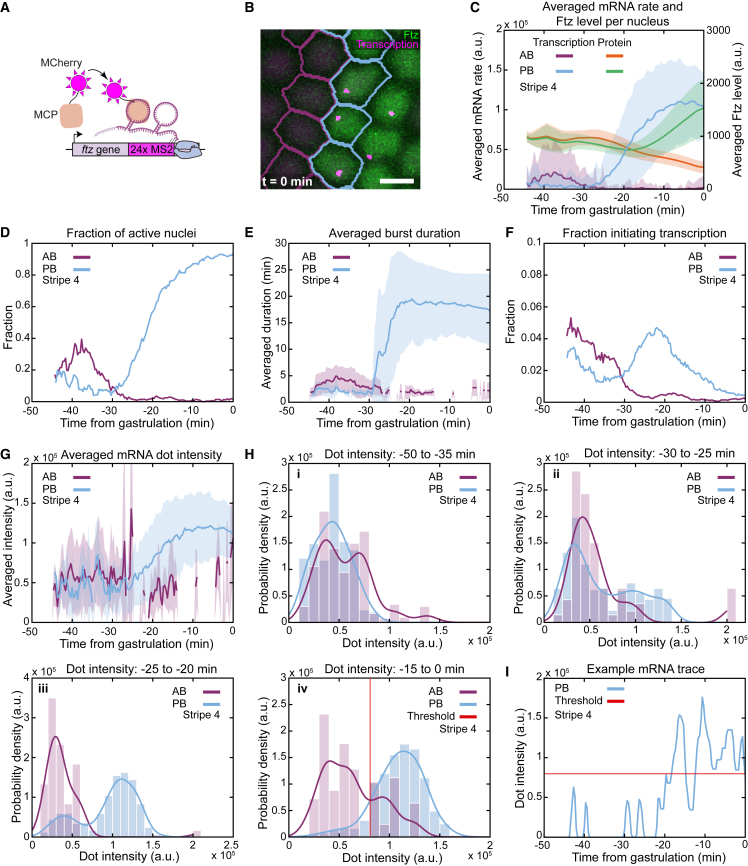
Transcription from the endogenous *ftz* gene occurs in two distinct modes (A) Approach to visualize transcription of the endogenous *ftz* gene in real time using MS2-MCP. (B) Example image of AB and PB nuclei in stripe 4, displaying endogenous *ftz* transcription together with Ftz protein. (C) Averaged mRNA rate per nucleus in boundary nuclei (left axis) and averaged Ftz levels (right axis). (D) The transcribing fraction of boundary nuclei. (E) The averaged burst duration in border nuclei displaying transcription. (F) The fraction of boundary nuclei initiating a transcription burst. (G) The averaged intensity of mRNA dots in boundary nuclei displaying transcription. (H) Histograms of all dot intensities occurring in boundary nuclei during 4 time periods. The instantaneous intensity of each dot is replaced by the average intensity of the transcription burst that it belongs to. Red line in (iv) indicates the intersection of the kernel distribution fit to the histograms of AB and PB nuclei. (I) Example PB transcription trace, with the red line indicating the threshold as determind in (Hiv). In (C), (E), and (G), mean over all included nuclei ± SD. In (D) and (F), fraction is calculated over all included nuclei; (H) shows kernel distribution fits to the histograms. Scale bars, 5 μm. See also Figures S2 and S3 and Video S6.

We used an allele of *ftz* that contained 24 copies of the MS2 stem loop sequence inserted into the endogenous 3′ UTR^24^^,^^25^^,^^26^^,^^27^ (Figure 2A). This made it possible to simultaneously measure transcription of endogenous *ftz* and follow Ftz protein levels in individual nuclei (Figure 2B; Video S6). We focus on stripe 4, but the general trends we observe are consistent across all examined stripes (Figure S2). We observed striking differences between the dynamics of *ftz* transcription and Ftz concentration in the boundary nuclei (Figure 2C). The average mRNA production rate per nucleus (i.e., spot intensity averaged over all visible nuclei) is similar in border nuclei up to around −30 min. However, after this time, transcription rates in PB nuclei start to exceed those in AB nuclei, which is 10 min before a similar trend is first seen in the Ftz traces. At gastrulation, the average levels of Ftz protein are about 4-fold higher in the PB nuclei, while the difference in averaged mRNA production rate is far greater at more than 50-fold.

Video S6. Zoom-in video of Fushi tarazu protein and endogenous-Ftz-MS2, related to Figure 2 Only mCherry signal is displayed in blue, with transcription dots accentuated in yellow. Scale bar is 20 μm.

Directly comparing the dynamics of Ftz protein and *ftz* transcription in border nuclei also reveals that the divergence in how *ftz* is transcribed cannot be caused by Ftz (Figure 2C, right axis). During the time when *ftz* transcription is specifically upregulated in PB nuclei and downregulated in the AB nuclei (−30 to −20 min), their average Ftz concentration is consistently decreasing. Ftz is a transcriptional activator, so if it is not inducing transcription in PB nuclei while at a higher level early, it most certainly cannot do so as its concentration decreases. In addition, during this time window the average Ftz levels are consistently lower in the PB versus the AB nuclei. If Ftz were responsible for the difference in transcription, its levels should be higher in the transcriptionally upregulated PB nuclei and not in the AB nuclei that are shutting down transcription. From this, we conclude that changes in Ftz concentration are not driving the differences in *ftz* transcription that define the distinct fate choice of AB and PB nuclei.

### Endogenous *ftz* transcription occurs in two distinct modes, characterized by burst duration and transcription rate

To understand exactly how transcription becomes different between the border nuclei, we examined four distinct features that define the average rate of mRNA production: fraction of active nuclei, average burst duration, the activation probability (fraction of nuclei initiating transcription), and average dot intensity. We observe that the fraction of active nuclei dominates the overall rate of mRNA production. Around −30 min, the fraction of transcribing PB nuclei begins to exceed that of AB nuclei and continues to increase, while transcription dissipates in the AB nuclei (Figure 2D). Before about −30 min, bursts are short (<5 min) in all boundary nuclei (Figure 2E). However, after this the bursts in PB nuclei begin to persist for much longer (up to ∼20 min), while those in AB nuclei remain short-lived. Similarly, the activation probability increases for PB nuclei from −30 to −20 min, while it dramatically falls in AB nuclei (Figure 2F). Taken together, this shows that from −30 to −20 min, differences in the fraction of active nuclei in the AB versus PB nuclei are driven by more nuclei activating and by having those nuclei transcribe for longer. Finally, we observed interesting changes in the average intensity of a transcription dot (Figure 2G), which we delve into next.

Up to about −25 min, the average dot intensity in border nuclei was very similar and relatively constant (Figure 2G). This trend continued for AB nuclei, but for PB nuclei average dot intensity started to increase until around −15 min. To understand how this increase occurred, we examined distributions of dot intensities (Figure 2H) and observed that, over time, these distributions split into two distinct groups. Early on the dots in both AB and PB nuclei had broadly overlapping intensity distributions, peaking at around 0.4 units (Figure 2Hi). As time progressed, a second distinct peak appeared in the PB intensity distribution, centered at around 1.1 units, although this did not occur for the AB dots (Figure 2Hii). For PB nuclei, this shift in the fraction of dots from the low-intensity group to the high-intensity group continued until few dots remained in the low-intensity group. In contrast, the distribution of dot intensities in AB nuclei remained relatively constant over time, without the appearance of a pronounced high-intensity peak (Figures 2Hiii and 2Hiv). Individual traces (Figure 2I) and their coefficients of variation (Figure S3B and 3C) showed that longer bursts initially fluctuate widely from lower to higher dot intensities, before settling after ∼10 min at a higher intensity value. First, taken together, this means that instead of dots gradually becoming brighter over time, the change in the average dot intensity was driven by a shift in the relative fraction of dots in each group. Second, it suggests that *ftz* is effectively transcribed in two discrete “modes.” The “low mode” is characterized by shorter bursts composed of dimmer dots, while the “high mode” is characterized by longer bursts with brighter dots (Figure S3D). The AB nuclei adopted the low mode throughout, while PB nuclei were initially in the low mode and after about −25 min started to move toward the high mode, first going through an intermediate phase of ∼10 min, characterized by the dot intensity fluctuating between low and high intensity, and finally reaching a high-intensity plateau at −15 min.

### Zebra and autoregulatory enhancers drive differential transcription burst dynamics

**Figure 3:**
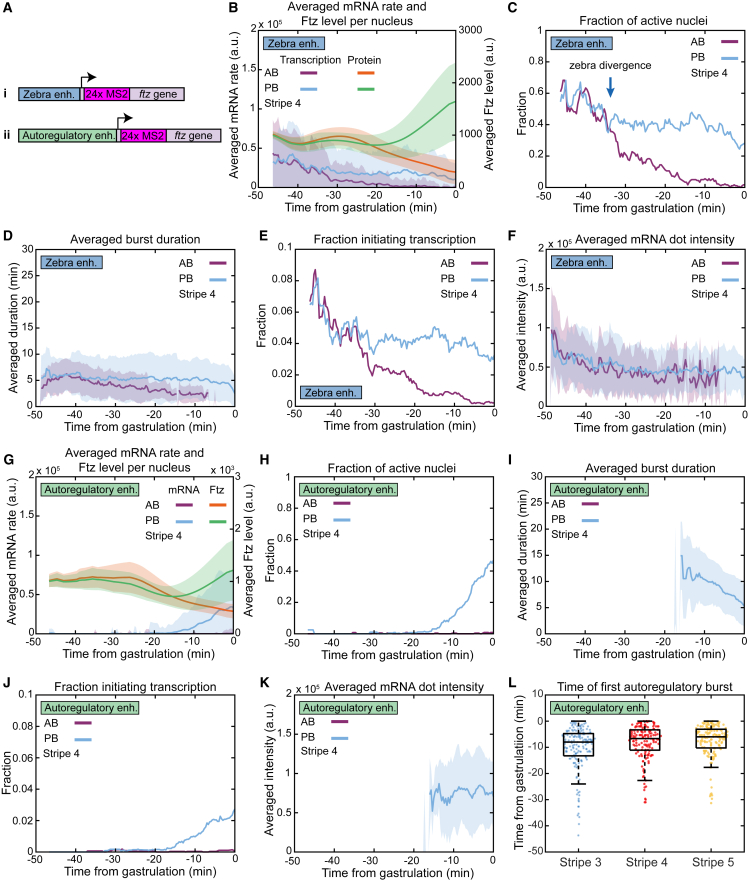
Zebra and autoregulatory enhancers drive differential transcription burst dynamics (A) Approach to visualize transcription of the (i) zebra and (ii) autoregulatory enhancer. (B and G) Averaged mRNA rate per nucleus in boundary nuclei (left axis) together with the averaged Ftz levels (right axis). (C and H) The transcribing fraction of boundary nuclei. (D and I) The averaged burst duration in boundary nuclei displaying transcription. (E and J) The fraction of boundary nuclei initiating a transcription burst. (F and K) The averaged intensity of mRNA dots in boundary nuclei displaying transcription. (L) Activation times of the autoregulatory enhancer in PB nuclei across stripes 3, 4, and 5. In (B), (D), (F), (G), (I), and (K), thick lines indicate the mean over all included nuclei, while shading is the standard deviation. In (C), (E), (H), and (J), the fraction is calculated over all included nuclei. See also Figures S3 and S4 and Videos S2, S3, S4, and S5.

To understand how the transcriptional dynamics of *ftz* is genetically encoded, we measured the transcriptional activity of the autoregulatory and zebra enhancers separately and compared this to endogenous transcription. We created transcriptional reporters for each enhancer, composed of the *ftz* transcription unit containing 24 repeats of the MS2 stem loop inserted into the 5′ intron^25^^,^^26^^,^^27^ (Figure 3A). These were integrated as single copies into the same genomic location (3L-65B2, *ftz* locus is at 3R-84A6), using the phiC31 site-specific integrase system.^28^ We then measured the transcriptional activity of each enhancer, while simultaneously following changes in endogenous Ftz protein levels. The reporters drove expression patterns consistent with what had been reported previously using fixed samples^5^^,^^13^^,^^15^ (Videos S2, S3, S4, and S5), but revealed some intriguing new dynamics.

Video S2. Full embryo video of Fushi tarazu protein and AutoregulatoryEnhancer-Ftz-MS2, related to Figure 3 Only mCherry signal is displayed in blue, with transcription dots accentuated in yellow. Scale bar is 20 μm.

Video S3. Zoom-in video of Fushi tarazu protein and AutoregulatoryEnhancer-Ftz-MS2, related to Figure 3 Only mCherry signal is displayed in blue, with transcription dots accentuated in yellow. Scale bar is 20 μm.

Video S4. Full embryo video of Fushi tarazu zebra-derived protein and ZebraEnhancer-Ftz-MS2, related to Figure 3 Only mCherry signal is displayed in blue, with transcription dots accentuated in yellow. Scale bar is 20 μm.

Video S5. Zoom-in video of Fushi tarazu zebra-derived protein and ZebraEnhancer-Ftz-MS2, related to Figure 3 Only mCherry signal is displayed in blue, with transcription dots accentuated in yellow. Scale bar is 20 μm.

The zebra enhancer initially drove broad expression but this became restricted to seven stripes that dissipated after gastrulation (Videos S4 and S5). At −40 min, the average mRNA production rate was similar in the PB versus AB nuclei, but by gastrulation it had become vastly different. This difference in transcription started at around −34 min, which is strikingly similar to when the divergence in transcription occurred at −30 min in endogenous *ftz* (compare Figures 2C and 3B). The few minutes’ time difference of this event in zebra versus endogenous is most likely due to the relative location of the MS2 loops. In the zebra reporter, these are located at the 5′ end, while in the endogenous construct they are in the 3′ UTR (Figures 2A and 3A). Changes in average mRNA production rate were driven largely by changes in the fraction of active nuclei (Figure 3C). The zebra-driven bursts in PB and AB nuclei remained short (<5 min) throughout, similar to the behavior of endogenous *ftz* before its transcription divergence occurs (Figures 2E and 3D). Therefore, the differences in the fraction of active nuclei (and, by extension, in the average mRNA rate) were due to the diverging activation probabilities in PB versus AB nuclei, starting from about −34 min (“zebra divergence”; Figure 3E). The activation probability of PB nuclei remained steady until about −15 min, when it started to decrease. Interestingly, we did not observe a substantial difference between the dot intensities of the emerging AB and PB nuclei (Figure 3F). From this, we conclude that the zebra enhancer is responsible for the transcription divergence observed around −30 min in the endogenous construct, which defines whether a nucleus becomes Ftz-high or Ftz-low. Furthermore, we can now associate the zebra enhancer with the population of short bursts composed of low-intensity dots in the endogenous transcription (Figures 3C–3F).

The autoregulatory enhancer construct activates transcription significantly later than zebra, but it appears in well-defined stripes that persist long after gastrulation (Videos S2 and S3). The average mRNA production rate starts to increase from about −15 min, and then only in the PB nuclei (Figure 3G). This increase is caused mostly by changes in the fraction of active nuclei and the activation probability (Figures 3H and 3J). Compared with zebra, this enhancer also produces bursts that are longer (up to ∼15 min; Figure 3I) and transcription dots that are brighter (Figure 3K). Similar to zebra, the average dot intensity remains relatively constant in time (Figure 3K). From this, we conclude that the longer bursts composed of brighter dots in the endogenous construct correspond to transcription driven by the autoregulatory enhancer. There is a delay of about 10 min between when we detect transcription from the autoregulatory enhancer reporter and when we see upregulation of transcription in endogenous *ftz*. We explore this “intermediate phase” further in the discussion.

### The autoregulatory enhancer is activated at a characteristic time, not at a specific Ftz concentration

**Figure 4:**
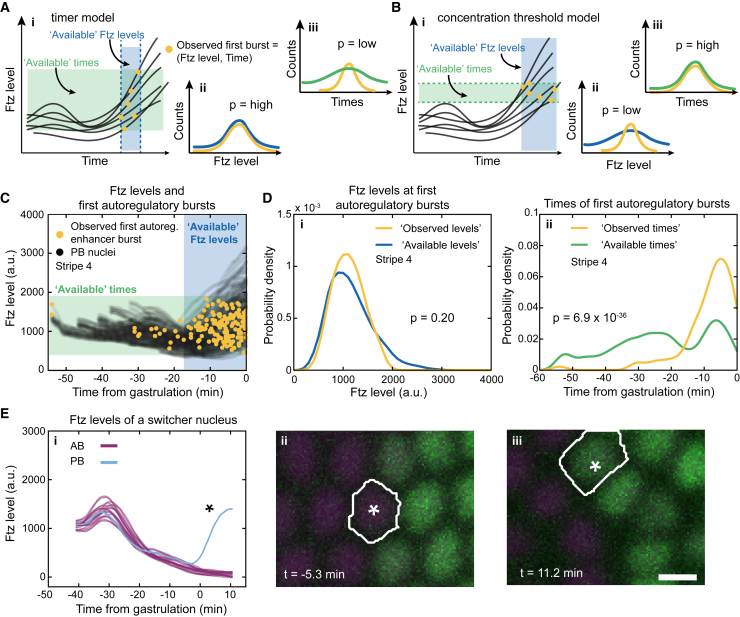
The autoregulatory enhancer is activated at a characteristic time and not when Ftz reaches a specific level (A) (i) In a timer model, transcription initiates during a defined time window (blue shading and dashed lines) and across a range of Ftz levels that correspond to those present within the time window (green shading). (ii) The potentially available Ftz levels (blue line) within the blue shading of (i) yields the same Ftz level distribution as observed (yellow line). (iii) The potentially available time points (green line) within the green shading of (i) yields a broader distribution than observed (yellow line). (B) (i) In a threshold model, transcription initiates at defined Ftz levels (green shading and dashed lines) and across a range of time points corresponding to the times at which those Ftz levels occur (blue shading). (ii) The potentially available Ftz levels (blue line) within the blue shading of (i) yield a broader Ftz distribution than observed (yellow line). (iii) The potentially available time points (green line) within the green shading of (Bi) yields the same time points distribution as observed (yellow line). (C) Scatterplot of Ftz levels in nuclei displaying transcription (stripe 4, 13 embryos). (D) (i) Observed Ftz levels at first autoregulatory enhancer burst (yellow) and potentially available Ftz levels (blue). (ii) Observed times of first autoregulatory enhancer burst (yellow) and potentially available time points (green). (E) (i) Examples of a PB nucleus in stripe 3 of an embryo whose Ftz levels mostly follow AB nuclei but rapidly increase close to gastrulation. (ii and iii) Images of the PB nucleus (white outline) near gastrulation (ii) and later when the Ftz levels have increased (iii). Two-sample Kolmogorov-Smirnov test was used for (D). Null hypothesis, observed and available values come from same distribution. Alternative hypothesis, available values tend to be smaller than observed values. For (Di), the test at 5% significance does not reject the null hypothesis. For (Dii), the test does reject the null hypothesis and accepts the alternative hypothesis. Scale bars, 5 μm. See also Figures S3 and S5.

We showed earlier that the initial differences in Ftz concentration do not specify whether a cell assumes a high- or low-Ftz state. Hence, the Ftz-dependent autoregulatory enhancer likely serves to merely maintain the earlier decision defined by zebra. To address how this maintenance is regulated, we needed to understand how the autoregulatory enhancer responds to Ftz. The prevailing model is that the enhancer is activated above a certain Ftz-concentration threshold^13^ (Figure 4B). Nevertheless, Figure 3L indicates that the enhancer was activated at similar times across multiple stripes, even though these stripes formed at different times (right axis of Figures 2C, S2A, and S2B). This is more consistent with a model in which the enhancer is activated after a specific time (Figure 4A).

To differentiate between a timer or a threshold model, we focused on nuclei as they initiated transcription of the autoregulatory enhancer for the first time. For each nucleus, we quantified when transcription started and what the Ftz concentration was at that time (Figures 4C and S5A). By combining this information with all the Ftz-concentration traces, we could generate “null” distributions composed of all potentially available Ftz and enhancer activation times. Then, we performed statistical tests to determine whether transcription initiated at a specific Ftz concentration or at a specific set of times (Figures 4A and 4B; see STAR Methods for details). This statistical approach makes it possible to use dynamic data on transcription and protein levels to test this hypothesis without having to make any additional assumptions. Our results show that the observed time distribution is different from the null time distribution (Figures 4Dii and S5Bii), while the observed Ftz level distribution is similar to the null levels distribution (Figures 4Di and S5Bi). Therefore, based on the available Ftz levels, the enhancer has the opportunity to activate at a much earlier time. Because it does not do this, the Ftz levels must not be of deciding importance for its activation. We saw this for each of the stripes used in these experiments (Figures S5C–S5F), which, taken together, strongly support a timer model over a threshold model for the activation of the autoregulatory enhancer. The timer model also predicts that, after the activation time, we should occasionally find nuclei with very low Ftz levels that become Ftz positive, which we do observe (Figure 4E).

### Timed pulses of ectopic Ftz support a time-controlled activation model

**Figure 5:**
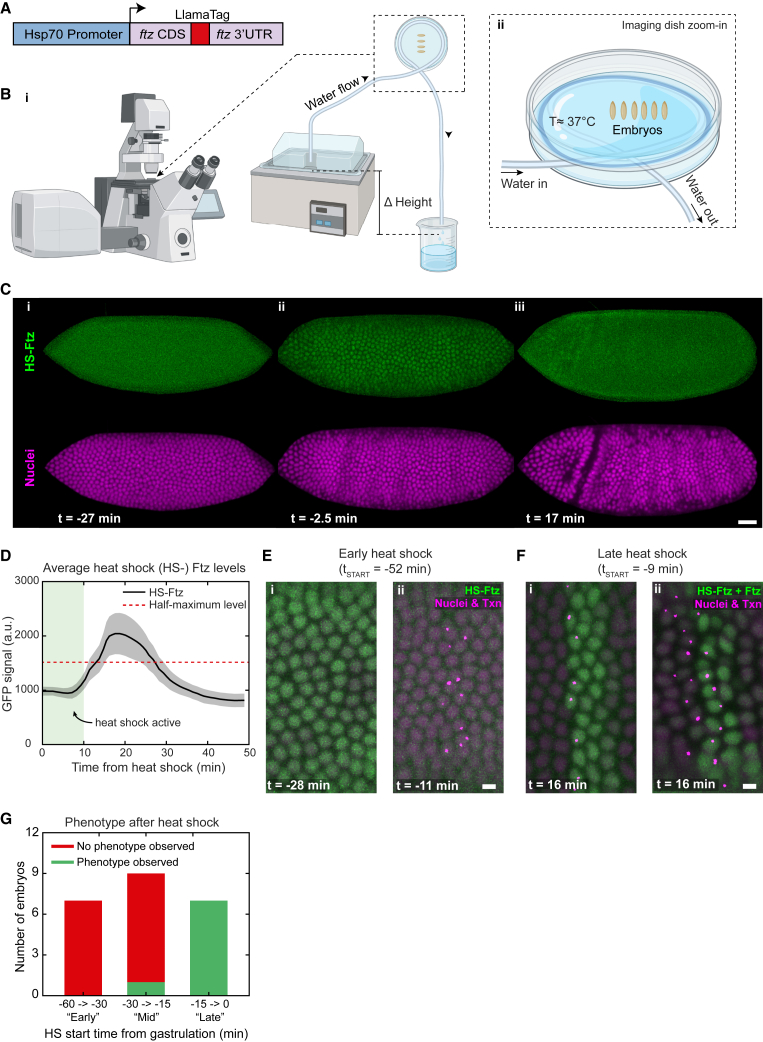
Induction of ectopic Ftz expression by heat shock only results in late ectopic autoregulatory enhancer activation (A) Construct used to induce heat shock. (B) Schematic of heat shock experiments. (i) Embryos are in a water-filled imaging dish and warm water flows from a heat bath through tubing heating the water in the dish by conduction. (ii) A zoom-in of the imaging dish. Created with BioRender.com. (C) Embryo undergoing heat shock. Green signal is Ftz produced by the heat shock (HS-Ftz). Magenta is nuclear marker. (i) Before heat shock. Only homogeneous background GFP is present. (ii) After heat shock the green signal is enriched inside the nuclei, indicating production of Ftz protein. (iii) Later, the green signal is again homogeneously distributed, indicating Ftz is degraded. (D) Raw GFP levels in nuclei of embryo shown in (C) (mean ± SD). Red line indicates the half-maximum of the mean heat shock levels. (E) An embryo heat shocked at an early time point (−52 min) does not show any ectopic autoregulatory enhancer expression. Green is Ftz produced by heat shock (HS-Ftz) and magenta dots are autoregulatory enhancer transcription. Two time points are shown as an example (i) −28 min, (ii) −11 min. (F) An embryo heat shocked at a late time point (−9 min) does show ectopic autoregulatory enhancer expression. Green signal is both endogenous Ftz and Ftz produced by heat shock (HS-Ftz), and magenta dots are autoregulatory enhancer transcription. (i) Control embryo without heat shock. (ii) Heat shocked embryo showing ectopic autoregulatory enhancer expression. (G) Number of embryos with and without ectopic autoregulatory expression (“phenotype observed” or “no phenotype observed”) as a function of the time at which the heat shock (HS) was started. Scale bars, 25 μm (C) and 5 μm (E and F).

To further test the timer model, we ectopically expressed Ftz at specific times before gastrulation to see whether this could induce transcription of the autoregulatory enhancer. Using a heat-shock-inducible promoter driving expression of a Ftz-LlamaTag fusion (Figure 5A), we developed a method to apply precisely timed heat shocks while concurrently imaging autoregulatory enhancer transcription and Ftz protein levels (Figure 5B). A relatively short and mild (∼10 min, 37°C) heat shock was able to induce a strong and ubiquitous pulse of ectopic Ftz protein. The short half-life of both the Ftz mRNA and protein ensured that the ectopic protein pulse was brief, lasting only about 15 min (Figures 5C and 5D).

When we ubiquitously expressed Ftz early (−60 to −30 min) we did not observe ectopic transcription of the autoregulatory enhancer. These embryos continued to develop and expressed the autoregulatory enhancer as per normal (Figures 5E and 5G). However, when Ftz was induced later (−15 to 0 min) we saw clear and consistent ectopic activation of transcription in AB nuclei and also other inter-stripe nuclei that usually do not express Ftz (Figures 5F and 5G). These data provide strong evidence that while the autoregulatory enhancer may need some Ftz to be activated, its activation is not regulated by exceeding a specific concentration of Ftz but rather through a timing-based mechanism.

### Ftz-dependent *engrailed* expression is also time controlled

**Figure 6:**
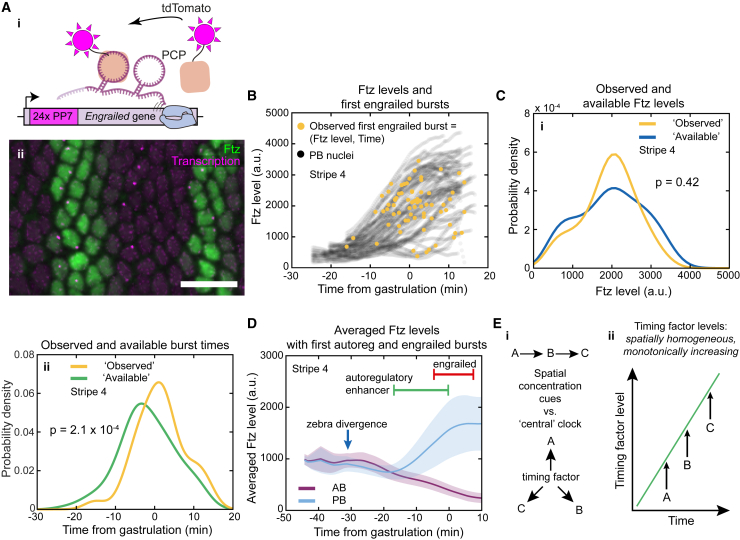
Ftz-dependent *engrailed* expression is also time controlled (A) (i) Visualizing *engrailed* transcription with PP7-PCP. (ii) Ftz protein and *engrailed* PP7 after gastrulation. (B) Ftz levels in transcribing nuclei (stripe 4, 5 embryos). (C) (i) Ftz levels at first *engrailed* transcription (yellow) and available Ftz levels (blue). (ii) Times of first *engrailed* transcription burst (yellow) and potentially available time points (green). (D) Mean Ftz level (± SD) in boundary nuclei in stripe 4, together with the annotation of zebra divergence and the time ranges (± SD) of autoregulatory enhancer and *engrailed* activation. (E) (i) Genes can be successively activated by regulating each other or through a timing factor acting as a central clock. (ii) A ubiquitous timing factor could successively activate genes (A, B, and C) through differential binding affinity of the timing factors to the enhancers of the genes. For (C), a two-sample Kolmogorov-Smirnov test was used, with the same null and alternative hypothesis as Figure 4D. Scale bars, 20 μm. See also Figures S3, S5, and S6.

To determine whether the timer model of Ftz activation may be relevant to other genes, we turned our attention to *engrailed*, which is a direct target of Ftz.^20^ To image its activation, we inserted PP7 tags into the endogenous gene using CRISPR^24^^,^^29^ (Figures 6Ai and 6Aii) and performed the same analysis as was done for the autoregulatory enhancer. We found that the distribution of observed times for *engrailed* activation was different from the available times distribution, while the set of the observed Ftz levels was similar to the available set of Ftz levels (Figures 6B, 6C, and S5G–S5J), mirroring the result of the autoregulatory enhancer. This provides additional support for the timer model over the threshold model for a second direct Ftz target, suggesting that this might be a more general way in which Ftz regulates its targets. This observation is also consistent with earlier work showing that the timing of *engrailed* activation does not change as the copy number of Ftz is altered.^11^ Interestingly, *engrailed* is activated about 10 min after the autoregulatory enhancer (Figure 6D), indicating that the precise activation time could be enhancer specific.

## Discussion

Our findings support a model where a cell’s destiny to express high or low levels of Ftz is defined by the zebra enhancer alone. This decision is then perpetuated by the autoregulatory enhancer. Something interesting is happening after the time that transcription in endogenous *ftz* is upregulated but before the autoregulatory enhancer reporter is activated. We propose that the changes in endogenous burst duration and dot brightness during this 10-min intermediate phase are consistent with a hand-off occurring between the two enhancers. We do not observe similar transition signatures in the reporters containing isolated enhancers, which suggests it is mediated by direct or indirect interaction between the two enhancers when they are present in *cis*. The seemingly premature activation of the autoregulatory enhancer in the endogenous context may be caused by an active zebra enhancer facilitating the autoregulatory enhancer to initiate before it can do so on its own. The large fluctuations between low- and high-intensity transcription values seen at the start of the intermediate phase are very much consistent with this view (Figures S3B and S3C).

We hypothesize that the timing control of the autoregulatory and *engrailed* enhancers is mediated by pioneer-like factors than modulate an enhancer’s ability to bind Ftz. It has been recently shown that the ubiquitously expressed transcription factors Caudal, Dichaete, and Odd-paired behave like simple timers within the early embryo.^17^^,^^30^^,^^31^^,^^32^ These three factors are sequentially expressed, bind to a diverse range of enhancers, and have pioneer-like activity through modulation of the chromatin state. In fact, Odd-paired has been shown to open the chromatin of the Ftz-dependent enhancers of *engrailed*,^31^ and the autoregulatory enhancer is known to be bound by both Dichaete and Odd-paired.^33^ The difference in activation timing between the autoregulatory enhancer and *engrailed* could be caused by predominant activation of *engrailed* through Odd-paired, which peaks later than Dichaete. Additionally, the timing difference might be related to differential affinity of the Odd-paired binding sites in the autoregulatory and engrailed enhancers. The concentration of Odd-paired peaks at gastrulation, but during the preceding half-hour window its concentration monotonically increases.^31^ Hence, the affinity of Odd-paired binding sites could define exactly when an enhancer becomes active during this time (Figure 6Eii).

Many processes, such as fate specification, morphogenesis, and cell migration, are happening in parallel during development. They all need to remain coordinated in both space and time, which is no small feat considering that they are driven by very different sets of genes, in cells that can be far apart. We speculate that regulating the relative activation times of various enhancers with global timing factors provides a means for ensuring this coordination. Such a “central clock” mechanism is distinct from a model of gene activation through progressive and sequential refinement of spatially distributed concentration cues (Figure 6E) but might provide additional robustness and coordination to the fast, non-equilibrium patterning processes in the embryo.

## STAR★Methods

### Key resources table

**Table:** 

REAGENT or RESOURCE	SOURCE	IDENTIFIER
**Experimental models: Organisms/strains**		

*D. melanogaster*: y[1] w[1118]; +; P{w[+mC]=vasa:eGFP }1,	Bothma et al.^21^	N/A
*D. melanogaster*: y[1] w[1118]; +; P{w[+mC]=nos:MCP-mCherry-NLS}	Bothma et al.^21^	N/A
*D. melanogaster*: y[1] w[1118]; +; P{w[+mC]=nos:tdTomatoPCP-NLS}	Fukaya et al.^34^	N/A
*D. melanogaster*: y[1] w[1118]; +; Ftz-LlamaTag	This study	N/A
*D. melanogaster*: y[1] w[1118]; en-PP7; +	This study	N/A
*D. melanogaster*: y[1] w[1118]; +; PBac{y[+mDint2] w[+mC]= Autoregulatory Enhancer:Ftz-MS2}VK00033	This study	N/A
*D. melanogaster*: y[1] w[1118]; +; PBac{y[+mDint2] w[+mC]=Zebra Enhancer:Ftz-MS2}VK00033	This study	N/A
*D. melanogaster*: y[1] w[1118]; +; Ftz-MS2	Lim et al.^24^	N/A
*D. melanogaster*: y[1] w[1118]; +; PBac{y[+mDint2] w[+mC]=Hsp70:FtzCDS-LlamaTag}VK00033	This study	N/A

**Recombinant DNA**		

See online repository for plasmid sequences	This study	https://benchling.com/bothmahubrecht/f_/1bKKlptD-ftz/

**Software and algorithms**		

Matlab	Mathworks	https://nl.mathworks.com/products/matlab.html
ImageJ/Fiji	Schindelin et al.^35^	https://fiji.sc/
TrackMate	Tinevez et al.^36^	https://imagej.net/plugins/trackmate/
Veed video compression	Veed.io	https://www.veed.io/tools/video-compressor
Drosophila Embryo Image Analysis Pipeline	This study and Bothma et al.^21^	Zenodo: https://doi.org/10.5281/zenodo.7787495

### Resource availability

#### Lead contact

Further information and requests for resources and reagents should be directed to and will be fulfilled by the lead contact, Jacques P. Bothma (j.bothma@hubrecht.eu).

#### Materials availability

All plasmids produced and fly lines generated in this paper will be shared by the lead contact upon request.

### Experimental model and subject details

The experimental model used in this study is *Drosophila melanogaster*. All individuals used in this study were embryos that were imaged as detailed below during the first 5 hours of development. Embryos were allowed to develop at room temperature and conditions unless otherwise stated. Embryo sex is not reported as it is not believed to influence any of the measurements reported here.

#### Regulatory landscape of the Ftz locus

Classic studies of the Ftz locus showed that it contains three main enhancers, namely the zebra and upstream enhancers that drive expression in the early embryo, and the neuro enhancer which drives expression in the developing nervous system (Figure S1).^12^ These studies also demonstrated that a minigene fragment of the *ftz* locus that includes only these three enhancers are able to make viable flies in a *ftz* null background showing these enhancers are sufficient to enable Ftz to define the body segments and build a functioning nervous system.^5^^,^^12^ In this study we refer to the upstream enhancer as an autoregulatory enhancer because this enhancer is regulated by the Ftz protein itself.^13^ Later studies^16^ identified three additional stripe-specific enhancers in the locus connected to each of the Ftz stripes, except for stripe 4. Since these are not required for rescue, they likely serve as “shadow” enhancers that facilitate patterning robustness in response to stress.^37^^,^^38^ We think it highly unlikely that other regions exist that regulate Ftz in the early embryo. The region of the genome that regulates Ftz is well defined because the *ftz* gene is flanked on either side by two highly effective and thoroughly characterized insulators^39^^,^^40^ (SF1 and SF2). Reporter gene studies and sequencing based contact assays have definitively shown that these insulators prevent the *ftz* gene from interacting with sequences outside this insulator-defined region.^40^^,^^41^^,^^42^ In addition, the chromatin state^33^ of the sequences between SF1 and SF2 in the early embryo has been mapped along with the binding of the transcription factor Zelda^43^ (which marks active enhancers). Taken together, these results indicate that there are no additional regulatory regions between SF1 and SF2 other than the aforementioned enhancers (Figure S1).

#### Fly Strains/Genotypes

The unpublished fly lines that were used in this study were generated by incorporating engineered transgenes into the genome of the *yw* fly strain, or by altering endogenous loci of the *yw* strain using CRISPR-Cas9 mediated homologous recombination. The cloning and transgenesis section details how each transgene was generated and genomically integrated, as well as how specific loci in the genome were edited using CRISPR-Cas9 mediated homologous recombination.

##### Ftz Protein and MCP-mCherry-NLS

To image the expression of Ftz protein in the early embryo, we performed fly crosses to combine the integrated transgene that encoded maternal eGFP, and maternal MCP-mCherry-NLS which served as a nuclear marker. The full genotype of these mothers was *yw; vasa>eGFP*; *nos*>MCP-mCherry-NLS/TM3. Virgin females of this line were collected, crossed in a cage with male flies that were homozygous for the Ftz locus and that had been tagged with the eGFP LlamaTag (*yw;+; Ftz-LlamaTag*). The resulting embryos were imaged. This ensured that the imaged embryos contained maternally deposited eGFP, MCP-mCherry-NLS, and the tagged Ftz locus. Furthermore, the effect of EGFP copy number on measured Ftz levels was tested by crossing *yw; vasa>eGFP*; *nos*>MCP-mCherry-NLS or *yw; vasa>eGFP/CyO*; *nos*>MCP-mCherry-NLS mothers to males containing *yw;+; Ftz-LlamaTag* or *yw;+; Ftz-LlamaTag*.

##### Ftz Protein and Fushi tarazu-MS2

To simultaneously image Ftz protein and transcription from the endogenous gene, we crossed mother flies that had the following genotype *yw; vasa>eGFP*; *nos*>MCP-mCherry-NLS with males of the genotype Fushi tarazu-MS2.^24^ To perform the imaging, we collected virgin females of the genotype *yw; vasa>eGFP*; *nos*>MCP-mCherry-NLS/Ftz-Llamatag. These were crossed in a cage with the males containing *yw;+;Ftz-MS2*. This ensured that the resulting embryos contained maternally deposited eGFP, MCP-mCherry-NLS, the tagged Ftz locus and Fushi tarazu-MS2.

##### Ftz Protein and AutoregEnhancer-Ftz-MS2

To simultaneously image Ftz protein and transcription from the AutoregulatoryEnhancer-Ftz-MS2 transgene, we created a recombinant chromosome containing both. This allowed us to generate a stable fly line with the following genotype: *yw;+;AutoregulatoryEnhancer-Ftz-MS2, Ftz-LlamaTag*. To perform the imaging, we collected virgin females of the genotype *yw; vasa>eGFP*; *nos*>MCP-mCherry-NLS and *yw; vasa>eGFP/CyO*; *nos*>MCP-mCherry-NLS. We used a mixture of females with 1 or 2 copies of eGFP, which were crossed in a cage with the males containing the recombinant chromosome, *yw;+;AutoregulatoryEnhancer-Ftz-MS2, Ftz-LlamaTag*. This ensured that the resulting embryos contained maternally deposited eGFP, MCP-mCherry-NLS, the tagged Ftz locus, and also the AutoregulatoryEnhancer-Ftz-MS2 transgene.

##### Ftz Protein and ZebraEnhancer-Ftz-MS2

To simultaneously image Ftz protein and transcription from that ZebraEnhancer-Ftz-MS2 transgene, we used the following approach. Using crosses, we generated mother flies that had the following genotype: *yw; vasa>eGFP/CyO*; *nos*>MCP-mCherry-NLS/ *Ftz-LlamaTag*. Virgin females were collected and crossed in a cage with males of the genotype *yw;+;ZebraEnhancer-Ftz-MS2*, and the resulting embryos were imaged. All the progeny had a copy of the *ZebraEnhancer-Ftz-MS2* transgenes and the 50% of embryos containing the *Ftz-LlamaTag* locus could easily be identified and selected on the microscope by looking for Ftz protein. This procedure ensured that the imaged embryos contained maternally deposited eGFP, MCP-mCherry-NLS, the tagged Ftz locus, and also the ZebraEnhancer-Ftz-MS2 transgene.

##### Ftz Protein and Engrailed-PP7

To image Ftz protein and engrailed transcription, we used fly crosses to generate male flies that contained both tagged loci with the following genotype: *en-PP7/CyO; Ftz-LlamaTag/TM3*. These were then crossed in a cage with virgin females that had a mixture of the following genotypes: *nos>tdTomatoPCP-NLS/CyO; vasa>eGFP/TM3* and *nos>tdTomatoPCP-NLS; vasa>eGFP*. The embryos that contained tagged Ftz could be identified by the presence of the protein pattern and those embryos were chosen for imaging. Only the subset of imaged embryos that later displayed engrailed transcription were then used for analysis. This ensured that the embryos that were imaged contained maternally deposited eGFP, PCP-tdTomato-NLS,^34^ the tagged Ftz locus, and also the PP7 tagged engrailed locus.

##### Ftz Protein, AutoregEnhancer-Ftz-MS2, HS-Ftz

To induce ectopic expression of FTZ and simultaneously image Ftz protein and transcription from the AutoregulatoryEnhancer-FTZ-MS2, mother flies that had the following genotype *yw; vasa>eGFP*; *nos*>MCP-mCherry-NLS were crossed with males with the following genotype yw;+;Hsp>FtzLlamaTag. To perform the imaging, virgin females of the genotype *yw; vasa>eGFP/+*; *nos*>MCP-mCherry-NLS/ Hsp>FtzLlamaTag were collected and crossed with males containing the following genotype: *yw;+;AutoregulatoryEnhancer-Ftz-MS2, Ftz-LlamaTag*. The resulting embryos were imaged, of which 50% of the progeny contained the *Hsp>FtzLlama*T*ag* transgene which could easily be identified and selected on the microscope by looking for ectopic Ftz expression 10 minutes after the start of the heatshock. This procedure ensured that the resulting embryos contained maternally deposited eGFP, MCP-mCherry-NLS, the tagged Ftz locus, the AutoregulatoryEnhancer-Ftz-MS2 transgene and also the Heatshock-ftz transgene.

### Method details

#### Cloning and Transgenesis

Details of the creation of the various transgenes can be found in the key resources table (under the entry ‘Recombinant DNA’). Briefly, the C terminal fusion of Ftz to the LlamaTag utilizing poly-glycine linker was based on a previously published mini-gene.^21^ For engrailed, 24 copies of PP7 loop sequences were inserted into the first intron in a poorly conserved region. Both donor constructs for CRISPR-Cas9 mediated homologous recombination were constructed using Gibson Assembly and the gRNAs were selected using the FlyCRISPR website; see sequence repository for further details. Injections were performed by BestGene into strain 54591. A 3xP3 driven dsRed cassette was added to the constructs for visual screening of CRISPR transformants. The autoregulatory, zebra and heat shock constructs were made in the pBPhi backbone using Gibson Assembly. For autoregulatory and zebra MS2 loops were placed in the endogenous Ftz intron as done in Bothma et al.^21^ The constructs were integrated on chromosome 3 using Bloomington strain 9750.

#### Embryo preparation for live imaging

Embryos were dechorionated using bleach, mounted between a semipermeable membrane (Biofolie, In Vitro Systems & Services) and a coverslip (1.5, 18 mm × 18 mm), and embedded in Halocarbon 27 oil (Sigma). Flattening of the embryos makes it possible to image a larger number of nuclei in the same focal plane without significantly impacting early development processes.

#### Laser scanning confocal microscopy

Embryos were imaged using a Zeiss LSM 980 confocal microscope. Confocal imaging on the Zeiss was performed using a Plan-Apochromat 40×/1.4NA oil immersion objective. GFP and the RFPs were excited with a laser wavelength of 488 nm (∼35 μW (0.3%) laser power) and 561 nm (∼20 μW (0.3-0.6%) laser power), respectively. Fluorescence was detected using the Zeiss QUASAR detection unit.

#### Imaging of embryos for heatshock experiments

Embryos were prepared using the previously described approach and mounted on a 27 mm Nunc Glass Base Dish (Thermo Fisher Scientific). Instead of using halocarbon oil, embryos were covered in a drop of room temperature water, after which a silicon tube with a diameter of 1.5 mm was placed in the dish as shown in Figure 5. After the dish was placed on the microscope and embryos found the heat shock was started by the addition of 5 ml of 37 °C water that covered the embryos and filled most of the dish and acted as a thermal bath for the embryos. In order to maintain the water in the dish at 37 °C over the course of ∼ 10 minutes, water flowed continuously through the tube from a water bath at higher temperature than 37 °C. The water that flowed through the tube did not directly contact the water in the dish, but passively transferred thermal energy through the silicon tube to the water in contact with the embryos. Through empirical tests it was established that with the flow rate used, maintaining the temperature of the water bath at 49 °C ensured the water in the dish stayed at a constant 37 °C. To halt the heat shock the source of the flow water was changed to room temperature which decreased the temperature of the water in the dish to room temperature over the course of several minutes.

### Quantification and statistical analysis

The sections below on ‘nuclei segmentation and tracking’ and ‘Ftz level and mRNA measurement’ are based on previous work.^21^

#### Determination of the gastrulation time

For each embryo a manual estimate of the gastrulation time is made (in the text referred to as ‘manual gastrulation’). This is done by finding the timepoint at which nuclei start displaying the first signs of movement due to gastrulation. In order to find a more objective measure of the gastrulation point, we looked at the movement of nuclei in the dorsal-ventral direction, which in our videos corresponded to the *y*-coordinate. For each nucleus in a particular embryo the timepoint of the last minimum or maximum in the *y*-coordinate over time was determined, which we coin the ‘local movement time’. After this timepoint the nucleus in question would display a single ‘directed’ movement until the end of the video. For each embryo, a kernel distribution was fitted (with automatic determination of kernel width by MATLAB) to the distribution of all local movement times, and the most common value was defined as the gastrulation time (Figures S1B and S1C).

#### Nuclei segmentation and tracking

The microscopy experiments resulted in videos with 5 dimensions: *xy* images in two channels (Ftz protein and mRNA/nuclear marker) at multiple *z*-planes and timepoints. The mRNA/nuclear channel functions as a reporter of transcription as well as a nuclear marker. CZI formatted files were imported into MATLAB, after which images were rotated to get the stripe pattern aligned in vertical direction. If the first few timepoints contained partially re-formed nuclei after nuclear division, these timepoints were removed from the video. To detect and segment the nuclei at each timepoint, a 10 slice sub-stack around the middle of the complete nuclear channel *z*-stack was maximum-intensity projected onto a single plane. Using a sub-stack for the projection was a compromise between maximizing detection of all nuclei, even if they moved out of plane, and reduction of motion-blur-induced deformation of nuclei after gastrulation. The MAX-projection was filtered using a Laplace of Gaussians filter with a user-chosen filter size appropriate to enhance features of a size similar to the nuclear diameter. A threshold was applied to the resulting image using Otsu’s method and the segmented nuclei were thickened by one pixel to smooth the edges. At this stage the nuclei were segmented but remained untracked through time. Tracking was done separately in ImageJ/Fiji^35^ using the TrackMate plugin^36^ with the Kalman tracking algorithm (LoG filter = 20 pixel object diameter; Initial search radius = 10 pixel; Search radius = 20 pixel; Max frame gap = 0 frames). The Kalman algorithm is especially suited to objects which move directionally through the frame. The tracks were imported into MATLAB and combined with the previously segmented nuclei, leading to segmented nuclei tracked through time. These segmented areas correspond to the central area of the nuclei, and in order to segment the full nucleus and also to include part of the cytoplasm in which the nucleus resides, the segmented nuclei were dilated until a plane-covering tiling was obtained. From this quasi-hexagonal tiling, the nearest neighbors of each nucleus were determined at every timepoint. Due to imaging conditions, tracking of nuclei in heat shock experiments for Figure 5D was not possible. Data points in this figure come from untracked nuclei visible in the field-of-view-at each timepoint.

#### Ftz level and mRNA measurement

To calculate the Ftz protein level in each nucleus, it is necessary to decide what *z*-slice is used for this measurement. Especially towards to end of nuclear cycle 14, some nuclei start to move in the *z*-direction, meaning that it is not possible to select a single *z*-slice which can be applied to all nuclei. Therefore, we selected per nucleus the brightest slice in the mRNA/nuclear marker channel, which corresponds to the *z*-plane in which the nucleus is in focus at that particular timepoint. The nuclear Ftz level was then calculated as the mean of the protein channel levels within the segmented nucleus area in three slices centered around the selected *z*-plane. mRNA spots were segmented in each *z*-plane by first applying a Laplace of Gaussians filter with a size appropriate to make dot-sized features stand out, followed by binarization with a user-chosen threshold. The choice of a particular value for this threshold was a compromise between maximizing the number of identified ‘real’ dots and minimizing the number falsely identified ‘spurious’ dots. A two-dimensional Gaussian function was fitted to the center of each detected dot, in order to subtract the local background. From a single mRNA dot, there might be signal in multiple *z*-planes, meaning that the dots belonging to a single mRNA spot must be clustered. From this cluster of dots, the brightest was chosen to represent the intensity of the mRNA spot in question. Finally, this information was linked to the expanded nucleus area in which the spot resides, leading to an mRNA signal per nucleus tracked over time.

#### Timepoint interpolation and smoothing

In order to have a consistent time interval between each time point across all embryos, we linearly interpolated the Ftz and mRNA signals to have a 20 s interval. Afterwards we averaged each nuclear Ftz trace with a 4 min moving window centered around each timepoint. Derivatives and second derivatives were also smoothed with a 4 min moving centered window after their calculation. Due to embryo movement, some nuclei moved out of the field-of-view during the video. Therefore, only those nuclei which were tracked from the start until the manual gastrulation time were taken into consideration for further analysis.

#### Boundary cell classification

To determine the set of AB and PB nuclei in each stripe, we used the stripe pattern at the manual gastrulation time as a basis. All visible nuclei were first sorted into two categories, ‘stripe’ and ‘non-stripe’ nuclei based on their measured Ftz level at the manual gastrulation time. The threshold for this sorting was determined by Otsu’s method, leading to the brightest nuclei to be classified as ‘stripe’ nuclei and the dimmest as ‘non-stripe’ nuclei (Figure S6E). Using this classification as a basis, the PB and AB nuclei were successively determined. The PB nuclei were defined as those ‘stripe’ nuclei, whose ‘non-stripe’ neighbors are all anteriorly located. Once the PB nuclei were identified, the AB nuclei were defined as those ‘non-stripe’ nuclei, which are anterior neighbors of the PB nuclei (Figure S6F). Finally, the PB and AB classified nuclei were assigned to a stripe number. This was done by thresholding a maximum-intensity projection (of an 8-slice sub-stack: 2 slices below to 5 slices above the center slice) at manual gastrulation time using Otsu’s method, resulting in a labelled binary image. Each nucleus was assigned the label of the nearest region in the labelled image (Figure S6G). Finally, the nuclei lists were restricted to those nuclei which are visible from the start until the manual gastrulation time. The aforementioned steps constituted the first automated guess of the AB and PB nuclei. Since the first guess usually was not entirely accurate, two rounds of refinement were performed. The first round consisted of two diagnostic steps, leading to a list of suggested nuclei which might be misclassified as ‘non-stripe’ nuclei. In the first diagnostic step, for each ‘non-stripe’ nucleus it was determined if its Ftz level increased *after* the manual gastrulation time, indicating that it might have been misclassified based on its Ftz level *at* manual gastrulation time. In the second diagnostic step, for each AB nucleus it was checked if it was more than one standard deviation brighter than the average AB nucleus at manual gastrulation time. The suggested nuclei from both steps (Figure S6Hi) were manually checked by inspecting the video after manual gastrulation time and the Ftz level traces (Figure S2Dii). If the suggestions were indeed found to be valid, the nuclei were moved from the ‘non-stripe’ list to the ‘stripe’ list, after which the algorithm, as detailed above, was repeated up to the stripe number assignment (Figure S6I). For the second round of refinement, only the second diagnostic step was performed, leading to another list of suggested nuclei (Figures S6Ji and S6Jii). In addition, the traces of the current AB and PB nuclei were checked for obvious errors, and added to the list of suggestions (Figure S6Jiii). This list of suggestions was again manually curated after which one last pass of the algorithm was done up to the stripe number assignment, resulting in the final determination of the AB and PB nuclei in each stripe (Figure S6K).

#### Background subtraction and motion correction

##### Endogenous, autoregulatory and zebra embryos

The endogenous and zebra embryos contain a single copy of GFP. However, due to experimental circumstances, the autoregulatory embryos were split into two populations: one subset of embryos were known to contain a single copy of GFP, while for the other subset it was not known if the embryo contained 1x or 2x GFP (phenotypic marker with CyO or without CyO, respectively). This means that it was not known what background value should be subtracted from the GFP channel. To determine the background level associated with each genotype, 6 background construct embryos with one and two copies of GFP were imaged (3 embryos per condition) and the average nuclear GFP level at the manual gastrulation time was determined for each embryo (Ftz_mean, gastrulation_). For one of the 2x GFP embryos, 3 nuclei were excluded because their Ftz level traces were obvious outliers. The background levels for 1x and 2x GFP were then calculated as the means of the obtained values of Ftz_mean, gastrulation_ for each genotype (Figure S1D). Next, 8 control embryos containing Ftz-LlamaTag and 1x or 2x GFP (4 embryos per condition) were imaged, and the median of the Ftz levels in the Anterior Boundary nuclei at the gastrulation time was determined for each embryo (Ftz_median, AB, gastrulation_, excluding outliers more than 1.5x Interquartile Range removed from the lower/upper quartile of the data). An upper threshold T_upper_ was calculated as the mean + standard deviation of obtained values of Ftz_median, AB, gastrulation_ for the control embryos containing 2x GFP. Similarly, a lower threshold T_lower_ was calculated as the mean - standard deviation of obtained values of Ftz_median, AB, gastrulation_ for the control embryos containing 1x GFP. Then we verified for the endogenous, zebra and 1x GFP autoregulatory embryos that their respective values of Ftz_median, AB, gastrulation_ (excluding outliers more than 1.5x Interquartile Range removed from the lower/upper quartile of the data) fell below the threshold T_upper_ (Figures S1E–S1G). Using this knowledge, we excluded all autoregulatory embryos with unknown GFP copy number for which was valid T_lower_ < Ftz_median, AB, gastrulation_ < T_upper_ (Figure S1H). The embryos with Ftz_median, AB, gastrulation_ > T_upper_ were assigned to 2x GFP and the embryos with Ftz_median, AB, gastrulation_ < T_lower_ were said to contain 1x GFP (Figure S1I). Furthermore, we verified that the distributions of Ftz levels in PB nuclei at gastrulation for embryos with either 1x or 2x GFP were not significantly different (Figure S1J). This means that the GFP levels are not limiting to our labelling method of Ftz.

##### Engrailed embryos

For the engrailed embryos it was not possible to follow the same background subtraction procedure as for the autoregulatory enhancer and zebra embryos (see above). This is due to the blue shifted emission of tdTomato relative to mCherry which meant there was a small amount of bleed though from the red channel to the green. Therefore the mean of the Ftz levels in the anterior neighbors of AB nuclei at the end of each engrailed video were used as the background value for that particular embryo (Figures S6A and S6B)

Most engrailed transcription happens around or after the gastrulation time (Figures 6B, S5G, and S5I). Since many nuclei move in the *z*-direction and out of plane in that time period, the Ftz levels in those nuclei might be altered due to distortions in the point spread function. In order to make statements about the Ftz levels at which *engrailed* transcription is activated, it is therefore necessary to compensate for this nuclear movement. This was done by correcting the Ftz values over time in each nucleus using the values of the mRNA/nuclear marker channel in that nucleus normalized to the initial timepoint. We verified the validity of this procedure by inspecting the eGFP and mRNA/nuclear marker levels in an engrailed background construct. As the eGFP and mRNA/nuclear levels in these constructs should be constant, any deviation from the initial value can be attributed to either bleaching or nuclear movement. By applying the correction as described above, a flat eGFP background level trace was recovered, compensating for the effects of movement after gastrulation and partially for the effects of bleaching before (Figures S7C and S7D).

#### mRNA trace treatment

To reduce the effects of intermittent detection of an mRNA spot (*e.g.*, the dot moving out of plane momentarily), dips in an mRNA trace of one or two time-frames were ‘filled up’, using a dilation followed by an erosion step. Both the dilation and the erosion used a three time-frame structuring element. This treatment was applied to all constructs.

#### Re-aligning traces to the gastrulation time

Since each video started at a variable time before gastrulation, it was necessary to align the traces according to an embryo-wide timepoint, namely the gastrulation time (see above, “determination of the gastrulation time”). This alignment can be done not only on Ftz traces and their derivatives, but also on mRNA traces. By default, the mRNA traces represent the intensity values at a particular timepoint (dot intensity). Additionally, these traces can be converted into traces of burst duration, fraction of active nuclei, and fraction of nuclei initiating transcription (burst activation probability). A burst duration trace is formed by replacing the dot intensity at a particular timepoint by the burst duration of the burst to which the dot at that timepoint belongs. To calculate the fraction of active nuclei we binarized each mRNA trace. A trace with the fraction of nuclei initiating transcription is obtained by marking only the first timepoint of each transcription burst. A coefficient of variation trace is obtained by calculating at each timepoint the square of the standard deviation in a moving 100 s window and dividing by the mean in that moving 100 s window. To achieve the trace re-alignment, the gastrulation time of each PB nucleus in a particular stripe of an embryo was set at t = 0 and the values of that nucleus’ Ftz, mRNA (or other) trace were shifted accordingly. AB neighbors of the PB nuclei were also aligned, by shifting the traces of the AB neighbors by the same amount as the PB nucleus in question, taking care to not count AB neighbors double. All PB and AB neighbor traces from the various embryos for the stripe in question were re-aligned, after which the stripe-level mean of the re-aligned traces was calculated. The number of nuclei participating in this mean varies across the re-aligned time axis, since each video was started at a different time before gastrulation. To avoid using a mean calculated from nuclei of a single embryo, a minimum number of participating nuclei of at least 25 was set for means of traces of Ftz levels, mRNA rate per nucleus, fraction of active nuclei, and fraction of nuclei initiating transcription (see Figure S3 for numbers of participating nuclei and embryos). In case of means of traces of mRNA dot intensity, burst duration and coefficient of variation the minimum number was set to 3 active dots participating in the mean. Additional smoothing in a 4 minute sliding time window was applied to the stripe-level mean for Ftz levels and the fraction of activating nuclei. For each re-aligned trace, except for fraction of active nuclei and fraction of nuclei initiating transcription, the standard deviation as a result of variation across contributing nuclei was also plotted.

#### Ftz level and timepoint shuffling scheme

For each embryo and in each stripe a list of PB nuclei displaying transcription was made. For each nucleus in this list, two numbers were obtained, namely the Ftz level at the start of the first mRNA burst and the timepoint of the start of that burst (relative to gastrulation). This generated, for each embryo and each stripe, a list of ‘observed’ Ftz levels and ‘observed’ timepoints (Figure S5A). Next, the ‘observed’ lists of timepoints and the Ftz levels were shuffled, meaning that a random permutation of the elements in the respective list was chosen. Then a list of ‘available’ timepoints was generated from the shuffled list of Ftz levels, and from the shuffled list of timepoints a list of ‘available’ Ftz levels. For the list of shuffled Ftz levels, the procedure was as follows. Each nucleus was assigned a shuffled Ftz value and it was checked at what timepoint that shuffled Ftz level occurred in the Ftz trace of the nucleus (within a 5% tolerance). If there were multiple possible timepoints, a random one was selected from the possibilities. If no possible timepoint was found, then no timepoint was registered for that nucleus. In this manner, list of ‘available’ timepoints was built for each permutation round. The process was repeated until the maximum number of permutations was reached, or 10000 times, whatever number was smaller, in the end resulting in an embryo-level list of ‘available’ timepoints. On the other hand, in the case of the shuffled timepoints, the procedure was as follows. Each nucleus was assigned a shuffled timepoint and this timepoint was applied to the Ftz trace of the nucleus in question, thereby generating a list of ‘available’ Ftz levels. This process was repeated until the total number of ‘available’ Ftz levels was comparable to the total number of previously generated ‘available’ timepoints, in the end resulting in an embryo-level list of ‘available’ Ftz levels (Figure S5B). This whole procedure was then applied to all embryos containing a particular stripe, resulting in four stripe-level lists: ‘observed’ Ftz level and timepoint lists, and lists of ‘available’ Ftz levels and timepoints, obtained from the shuffled timepoints and Ftz levels, respectively (Autoregulatory enhancer: Figures 6F, S5D, and S5F; Engrailed: Figures 7E, S5H, and S5J). Next, a statistical test was performed to check if the observed and ‘available’ lists were the different or not. Since the distributions are non-normal, the non-parametric two-sample Kolmogorov-Smirnov test was chosen (at the 5% significance level), which is both sensitive to location and shape of a distribution. For the null-hypothesis we stated that the ‘observed’ and ‘available’ distribution (for either Ftz level or timepoints) were drawn from the same continuous distribution. The alternative hypothesis was that the ‘available’ distribution had smaller values (*i.e.*, earlier timepoints or lower Ftz levels) than the ‘observed’ distribution.

#### Histograms, kernel distribution fits, boxplots

For most histograms (except Figures 2H, S1C, S1J, S2I, and S2J, with bin widths of 10000, 1, 250, 10000 and 10000, respectively) the bin width was automatically decided using the Freedman-Diaconis rule, which is less sensitive to outliers in data, and is thus suited for more irregular shaped distributions. In those cases that multiple histograms or kernel distribution fits were plotted in the same figure, the bin width or kernel width was decided on the distribution with the lowest number of counts. In cases that kernel distribution fits and histograms were shown together, the kernel width and the bin width were chosen to be equal (except in Figure S1C, where the bin width and the kernel width were chosen automatically by MATLAB). In boxplots, the bottom and top edges of the box denote the 25^th^ and 75^th^ percentiles of the data, respectively and the line inside the box indicates the median, the whiskers extend to the minimum and maximum of the data not considered outliers. A data point is considered an outlier if it lies more than 1.5 times the interquartile range from the bottom or top of the box.

#### Heat shock analysis

Embryos were divided in three groups (‘early’ -60 min to -30 min; ‘mid’-30 min to -15 min; and ‘late’ -15 min to 0 min) based on the time difference between the start of the heat shock and gastrulation time. Embryos were manually checked for the presence of a transcriptional phenotype. This phenotype included overall gain of ectopic transcription in any nucleus in the early heat shocked embryos, while for the late heat shocked embryos this only included the gain of ectopic transcription in the majority of anterior neighbors of the AB nuclei.

#### Trace selection and image treatment

For endogenous construct, zebra enhancer, and autoregulatory enhancer embryos, the Ftz and mRNA levels, and associated quantities such as starting times of the first mRNA burst, are considered unreliable after gastrulation due to nuclear movement, and are therefore disregarded in all figures. In case of engrailed embryos, a nuclear movement correction is performed, meaning that for engrailed embryos these values and quantities are included. Any additional trace selection and image treatment is detailed below.

Figures 1C and 1D: Maximum intensity projection of green (MaxG) and red channel (MaxR). Gaussian blur applied to MaxR (MaxRBlur). MaxRBlur and MaxG were combined, and relative intensities of channels were adjusted.

Figure 1D: Maximum intensity projection of green (MaxG) and red channel (MaxR). Gaussian blur applied to MaxR (MaxRBlur). MaxRBlur and MaxG were combined, and relative intensities of channels were adjusted.

Figure 2B: Maximum intensity projection was made of green (MaxG) and red channel (MaxR). Gaussian blur was applied to MaxR and the image was normalized to its maximum value (MaxRBlur). Separately, a LoG filter of size comparable to transcription dots was applied to all slices of the red channel, and subsequently a max projection was done (Dots). The Dots image was binarized to retain only the dots (DotsMask). Using DotsMask was applied to MaxRBlur and subtracted from MaxRBlue to remove dots (MaxRBlurNoDots). Then MaxRBlurNoDots was normalized and the dots were re-added by combing MaxRBlurNoDots with DotsMask, while adjusting their relative intensities (RedFinal). Lastly, the MaxG was normalized to its maximum value, and subsequently combined with the RedFinal image. A crop of the full image was made for display purposes.

Figure 3L: Exclude PB nuclei without transcription before gastrulation.

Figures 4C, 4D, and S5A–S5D: Exclude PB nuclei without transcription before gastrulation.

Figure 4Ei: PB nuclei that increased their Ftz levels after gastrulation time were manually selected from all available traces. Then it was verified that the nuclear tracking of these nuclei was accurate to exclude an increase of Ftz due to tracking error.

Figures 4Eii and 4Eiii: Maximum intensity projection of green (MaxG) and red channel (MaxR). Gaussian blur applied to MaxR (MaxRBlur). MaxRBlur and MaxG were combined, and relative intensities of channels were adjusted.

Figure 5C: Due to imaging conditions, only a selection of z-slices was used, namely the slices from which the vitelline membrane could be satisfactorily removed. Maximum intensity projection of green (MaxG) and red channel (MaxR). Gaussian blur applied to MaxR (MaxRBlur).

Figure 5D: Due to imaging conditions, exclude badly segmented nuclei by removing at each timepoint nuclei whose segmented area and expanded area (see above) were 2x median absolute deviations (MAD) removed from the median area value. Lastly, also remove nuclei at each timepoint with anomalous fluorescence levels more than 3x MAD removed from the median intensity value.

Figures 5E and 5F: Due to imaging conditions, only a selection of z-slices was used, namely the slices for which the presence of vitelline membrane was not too hindering, while making sure that no transcription dots were excluded. Maximum intensity projection was made of green (MaxG) and red channel (MaxR). Gaussian blur was applied to MaxR and the image was normalized to its maximum value (MaxRBlur). Separately, a LoG filter of size comparable to transcription dots was applied to all slices of the red channel, and subsequently a max projection was done (Dots). The Dots image was binarized to retain only the dots (DotsMask). Using DotsMask was applied to MaxRBlur and subtracted from MaxRBlue to remove dots (MaxRBlurNoDots). Then MaxRBlurNoDots was normalized and the dots were re-added by combing MaxRBlurNoDots with DotsMask, while adjusting their relative intensities (RedFinal). Lastly, the MaxG was normalized to its maximum value, and subsequently combined with the RedFinal image. A crop of the full image was made for display purposes.

Figure 6A: Maximum intensity projection was made of green channel (MaxG). A LoG filter of size comparable to transcription dots was applied to all slices of the red channel, and subsequently a max projection was done (Dots). The MaxG and Dots image were combined, while adjusting their relative intensities. A crop of the full image was made for display purposes.

Figures 6B, 6C, and S5G–S5J: Exclude PB nuclei without transcription.

Figure 6D: Exclude PB nuclei without transcription before gastrulation for autoregulatory enhancer annotation. Exclude PB nuclei without transcription for engrailed annotation. Exclude Ftz levels more than 10 min after gastrulation.

Figure S3D: Exclude nuclei without transcription.

Figure S1D: Only use nuclei with complete trace up to manual gastrulation.

Figure S6 (images): Maximum intensity projection of green (MaxG) and red channel (MaxR). Gaussian blur applied to MaxR (MaxRBlur). MaxRBlur and MaxG were combined, and relative intensities of channels were adjusted.

Video S1: Maximum intensity projection of green (MaxG) and red channel (MaxR). Gaussian blur applied to MaxR (MaxRBlur). MaxRBlur and MaxG were combined, and relative intensities of channels were adjusted.

Video S2. Full embryo video of Fushi tarazu protein and AutoregulatoryEnhancer-Ftz-MS2, related to Figure 3, Video S3. Zoom-in video of Fushi tarazu protein and AutoregulatoryEnhancer-Ftz-MS2, related to Figure 3, Video S4. Full embryo video of Fushi tarazu zebra-derived protein and ZebraEnhancer-Ftz-MS2, related to Figure 3, Video S5. Zoom-in video of Fushi tarazu zebra-derived protein and ZebraEnhancer-Ftz-MS2, related to Figure 3: Only the signal from red channel was used. Signal from the vitelline membrane was removed in each z-slice (if applicable). Maximum intensity projection of red channel followed by Gaussian blur (NucBlur). In parallel, LoG filter (filter size appropriate to enhance dots) was applied to each red channel slice, followed by maximum intensity projection (Dots). For the video the nuclei are shown in Blue and the transcription dots are shown in Yellow. The Dots image was gamma-corrected and intensity scaled to reduce LoG filter artifacts, yielding the Yellow channel. The gamma-corrected and scaled Dots image was subtracted from an intensity scaled NucBlur image, yielding the Blue channel. The combined Yellow and Blue images were automatically rotated to obtain the correct orientation, as needed.

## Data Availability

• All imaging data reported in this paper will be shared by the lead contact upon request. • All original code has been deposited at Zenodo and is publicly available as of the date of publication. DOIs are listed in the key resources table. • Any additional information required to re-analyze the data reported in this paper is available from the lead contact upon request.

## Supplemental information

Document S1. Figures S1–S6

Document S2. Article plus supplemental information
