# Supplementary material for: Precisely timed regulation of enhancer activity defines the binary expression pattern of Fushi tarazu in the Drosophila embryo
Source: Curr Biol. 2023 Jul 24;33(14):2839–2850.e7. doi: 10.1016/j.cub.2023.04.005 (PMC10373528; doi:10.1016/j.cub.2023.04.005)
Supplement: Document S1. Figures S1–S6 [file mmc1.pdf]

Current Biology, Volume *33*

Supplemental Information

Precisely timed regulation of enhancer activity defines the binary expression pattern of Fushi tarazu in the *Drosophila* embryo

Anthony Birnie, Audrey Plat, Cemil Korkmaz, and Jacques P. Bothma

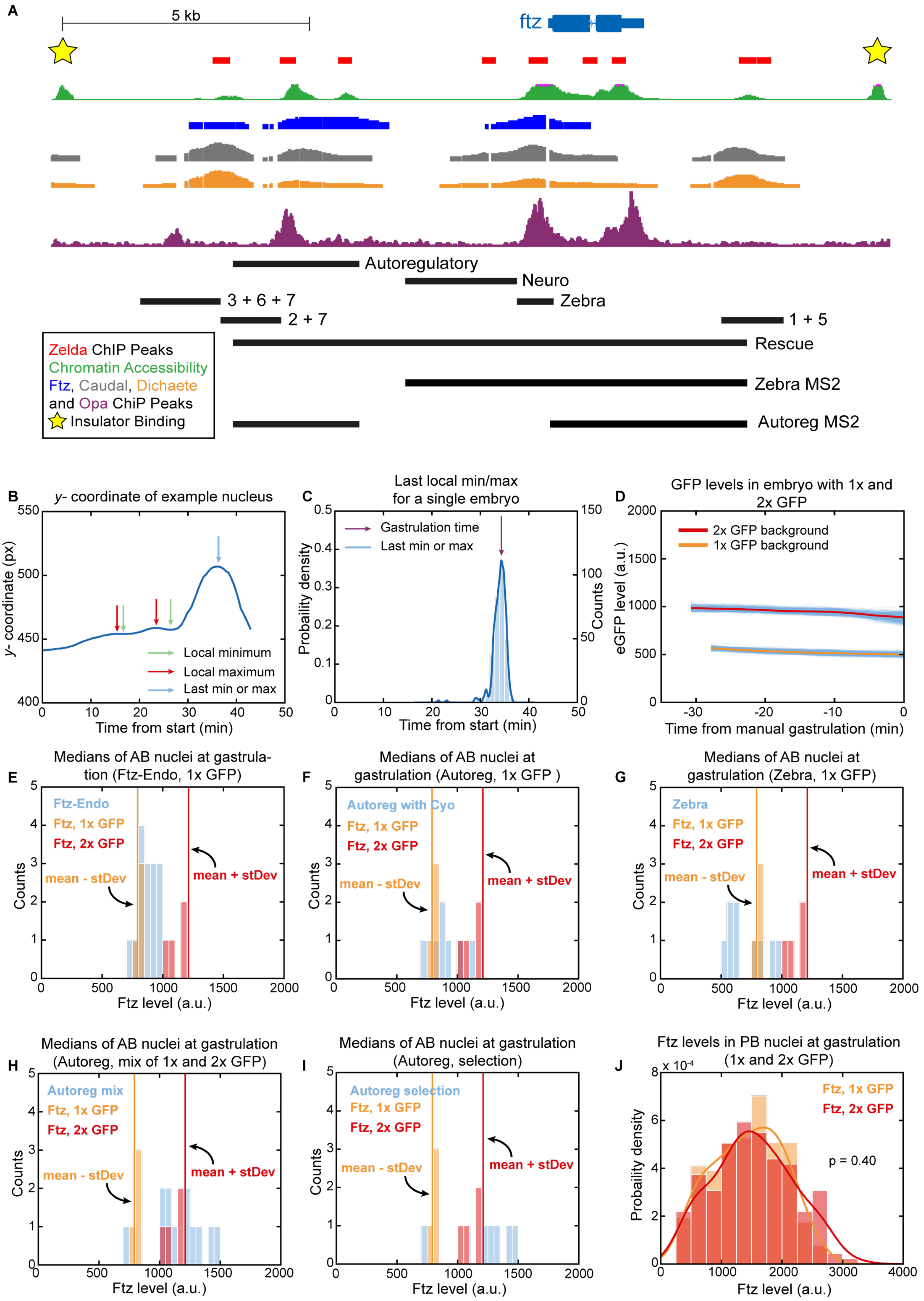
A
5 kb
ftz
Autoregulatory
Neuro
3 + 6 + 7
Zebra
2 + 7
1 + 5
Rescue
Zebra MS2
Autoreg MS2
Zelda ChIP Peaks
Chromatin Accessibility
Ftz, Caudal, Dichaete
and Opa ChiP Peaks
Insulator Binding
B
y- coordinate of example nucleus
y- coordinate (px)
Local minimum
Local maximum
Last min or max
Time from start (min)
C
Last local min/max
for a single embryo
Gastrulation time
Last min or max
Probability density
Counts
Time from start (min)
D
GFP levels in embryo with 1x and 2x GFP
2x GFP background
1x GFP background
eGFP level (a.u.)
Time from manual gastrulation (min)
E
Medians of AB nuclei at gastrulation (Ftz-Endo, 1x GFP)
Ftz-Endo
Ftz, 1x GFP
Ftz, 2x GFP
mean - stDev
mean + stDev
Counts
Ftz level (a.u.)
F
Medians of AB nuclei at gastrulation (Autoreg, 1x GFP)
Autoreg with Cyo
Ftz, 1x GFP
Ftz, 2x GFP
mean - stDev
mean + stDev
Counts
Ftz level (a.u.)
G
Medians of AB nuclei at gastrulation (Zebra, 1x GFP)
Zebra
Ftz, 1x GFP
Ftz, 2x GFP
mean - stDev
mean + stDev
Counts
Ftz level (a.u.)
H
Medians of AB nuclei at gastrulation (Autoreg, mix of 1x and 2x GFP)
Autoreg mix
Ftz, 1x GFP
Ftz, 2x GFP
mean - stDev
mean + stDev
Counts
Ftz level (a.u.)
I
Medians of AB nuclei at gastrulation (Autoreg, selection)
Autoreg selection
Ftz, 1x GFP
Ftz, 2x GFP
mean - stDev
mean + stDev
Counts
Ftz level (a.u.)
J
Ftz levels in PB nuclei at gastrulation (1x and 2x GFP)
x 10-4
Ftz, 1x GFP
Ftz, 2x GFP
p = 0.40
Probability density
Ftz level (a.u.)

Figure S1. Regulatory landscape of the *ftz* locus and determination of gastrulation time and background subtraction for endogenous, autoregulatory, and zebra enhancer embryos, Related to Figure 1 and STAR-methods.

(**A**) Schematic illustrating the *ftz* locus along with regions of known regulatory activity, a map of chromatin accessibility, ChiP peaks for Zelda, Ftz, Caudal, Dichaete, and (Odd-paired) Opa, and the location of the insulators [S1–S3]. The region capable of rescuing a *ftz*-null mutant as a minigene is also shown together with the sequences used in the autoregulatory and zebra reporters used in this study. (**B**) The *y*-coordinate, roughly corresponding to the dorsal-ventral axis, of an example nucleus. Local minima and maxima are annotated with arrows. The time coordinate of the last local minimum or maximum corresponds to the 'local movement time'. (**C**) The distribution of "local movement times" of all nuclei in a single embryo. Line corresponds to kernel distribution fit of the histogram, and the most common value is annotated as the embryo-wide movement time (gastrulation time). (**D**) Background eGFP level relative to manual gastrulation for the 1x and 2x GFP background construct. Individual traces are shown including the mean in bold. (**E**), (**F**), (**G**) Medians of Ftz levels in AB nuclei at gastrulation time in embryos for which the GFP copy number was phenotypically known to be 1x GFP (blue bars): endogenous, autoregulatory (1x GFP), zebra, respectively. Control embryos with 1x and 2x GFP are shown as a reference in yellow and red bars, respectively. (**H**) Medians of Ftz levels in AB nuclei at gastrulation time in autoregulatory embryos (blue bars) for which the GFP copy number was unknown (1x or 2x GFP). Control embryos with 1x and 2x GFP are shown as a reference in yellow and red bars, respectively. (**I**) Medians (in blue bars) of Ftz levels in AB nuclei at gastrulation time in autoregulatory embryos after removing embryos whose median lies between the yellow and red line. (**J**) Ftz levels in PB nuclei at gastrulation time in 1x and 2x GFP embryos, indicating that after background subtraction there is no statistically significant difference between the two distributions. Yellow and red lines are kernel distribution fits to the data. Two-sample Kolmogorov-Smirnov test was used. Null-hypothesis = 1x GFP and 2x GFP values come from same distribution. Alternative hypothesis = 1x GFP and 2x GFP values come from different distribution. In panels E-**i**, the yellow line is the mean – standard deviation of the 1x GFP control embryos (yellow bars), whereas the red line is the mean + standard deviation of the 2x GFP control embryos (red bars).

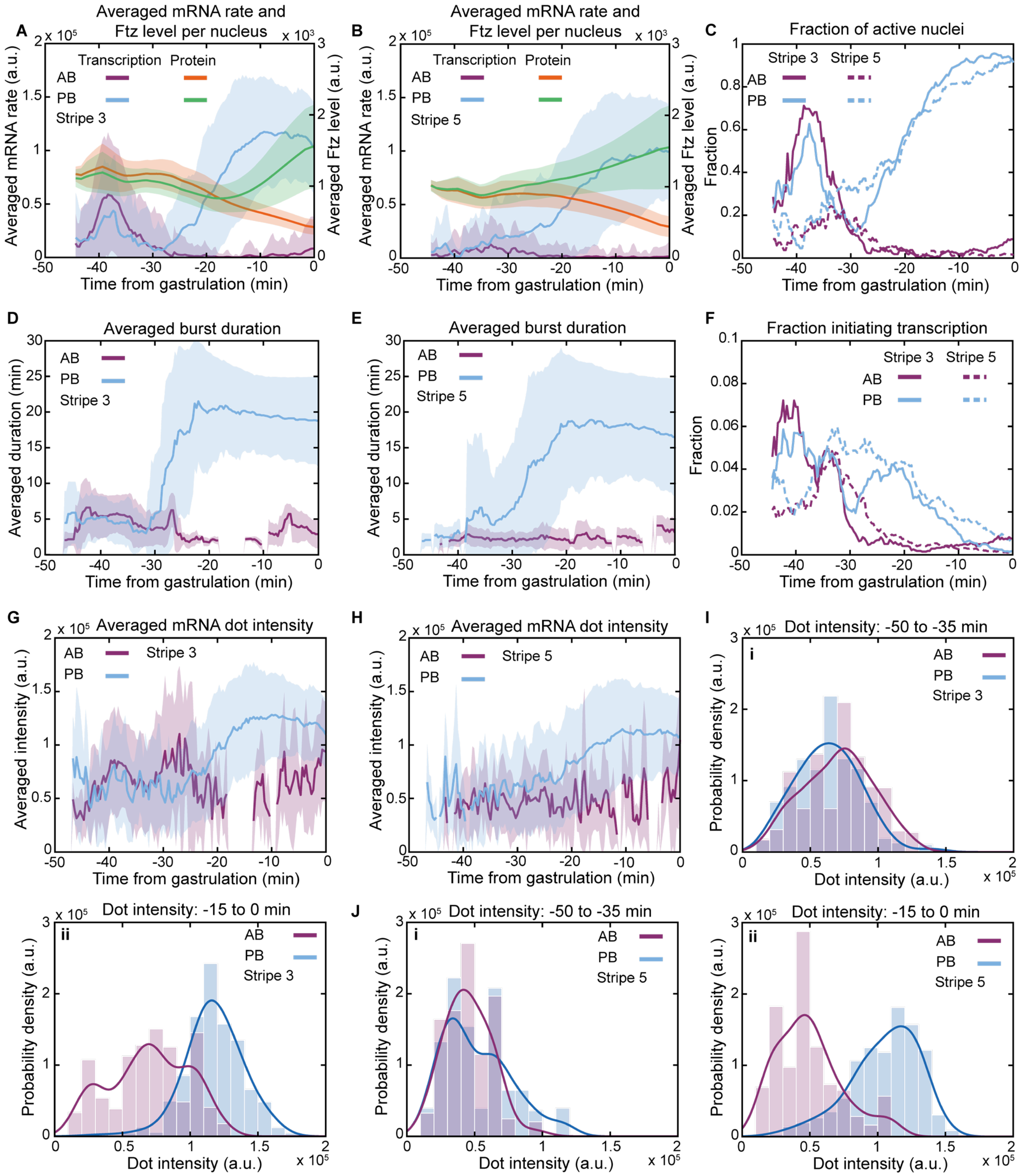

Figure S2. Transcription of the endogenous *ftz* gene in stripe 3 and 5, Related to Figure 2.
(**A**) (Stripe 3), (**B**) (Stripe 5) Averaged mRNA rate per nucleus in AB and PB nuclei (left axis) together with the averaged Ftz levels in those nuclei (right axis). (**C**) The transcribing fraction of AB and PB nuclei in Stripe 3 (straight line) and Stripe 5 (dashed line). (**D**) (Stripe 3)**,** (**E**) (Stripe 5) The averaged burst duration in AB and PB nuclei displaying transcription. (**F**) The fraction of AB and PB nuclei initiating a transcription burst in Stripe 3 (straight line) and Stripe 5 (dashed line). (**G**) (Stripe 3), (**H**) (Stripe 5) The averaged intensity of mRNA dots in AB and PB nuclei displaying transcription. (**I**) (Stripe 3), (**J**) (Stripe

5) Histograms of all dot intensities occurring in AB and PB nuclei during 2 time periods. The instantaneous intensity of each dot is replaced by the average intensity of the transcription burst that it belongs to. -**i** -50 min to -35 min. -**ii** -15 min to 0 min. In panels A, B, D, E, G and H thick lines indicate the mean over all included nuclei, while shading is the standard deviation. In panels C and F, the fraction is calculated over all included nuclei. In panels I and J, the thick lines indicate kernel distribution fits to the histograms.

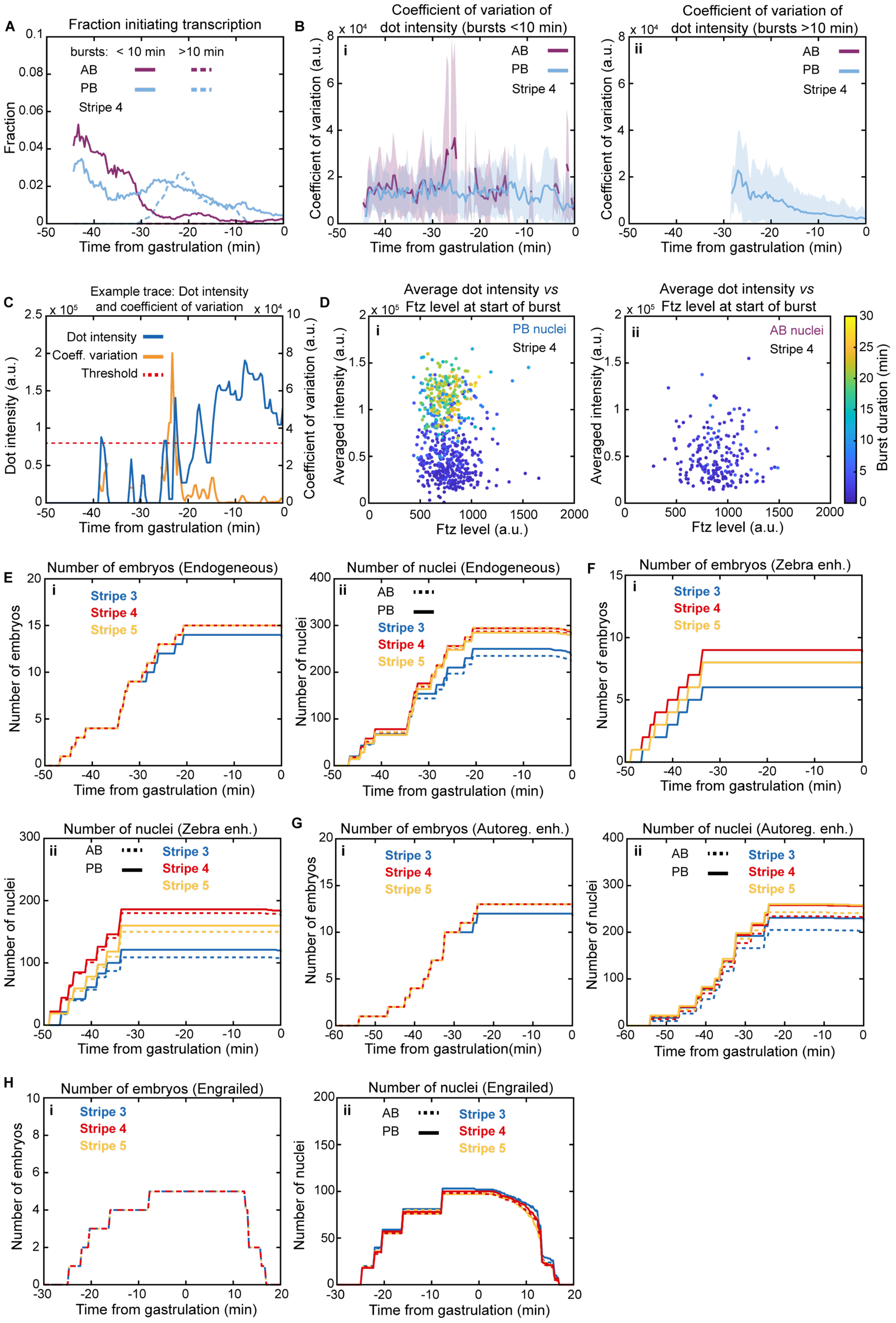
A
Fraction initiating transcription
bursts: < 10 min >10 min
AB
PB
Stripe 4
Fraction
Time from gastrulation (min)
B
Coefficient of variation of dot intensity (bursts <10 min)
i
Coefficient of variation (a.u.)
x 10^4
Coefficient of variation of dot intensity (bursts >10 min)
ii
Time from gastrulation (min)
C
Example trace: Dot intensity and coefficient of variation
x 10^5
x 10^4
Dot intensity
Coeff. variation
Threshold
Dot intensity (a.u.)
Coefficient of variation (a.u.)
Time from gastrulation (min)
D
Average dot intensity vs Ftz level at start of burst
i
PB nuclei
Stripe 4
Averaged intensity (a.u.)
Ftz level (a.u.)
ii
AB nuclei
Burst duration (min)
E
Number of embryos (Endogeneous)
Stripe 3
Stripe 4
Stripe 5
Number of embryos
Number of nuclei (Endogeneous)
Number of nuclei
F
Number of embryos (Zebra enh.)
Number of nuclei (Zebra enh.)
G
Number of embryos (Autoreg. enh.)
Time from gastrulation(min)
Number of nuclei (Autoreg. enh.)
H
Number of embryos (Engrailed)
Number of nuclei (Engrailed)

Figure S3. Transcription of the endogenous *ftz* gene (stripe 4) and embryo and nuclei numbers for endogenous, zebra, autoregulatory, and engrailed constructs, Related to Figure 2, 3, 4 and 6. (**A**) The fraction of AB and PB nuclei initiating a transcription burst in Stripe 4, for burst shorter (straight line) and longer (dashed line) than 10 min. (**B**) The averaged coefficient of variation for transcription bursts in AB and PB nuclei in Stripe 4: **-i** shorter than 10 min, and **-ii** longer than 10 min. (**C**) An example trace of dot intensity (left axis, blue line) in a PB nucleus and the coefficient of variation (right axis, orange line). The dashed red line is the threshold as determined in panel H-**iv** of figure 2. (**D**) Average dot intensity in a burst *vs.* the Ftz level at the start of a burst nuclei of Stripe 4. Color of the dot indicates burst duration. -**i** PB nuclei, and -**ii** AB nuclei. (**E**)**.** (endogenous), (**F**) (zebra)**,** (**G**) (autoregulatory)**,** (**H**) (engrailed) -**i** Number of participating embryos in Stripe 3, 4, and 5. (**E**) (endogenous)**,** (**F**) (zebra)**,** (**G**) (autoregulatory), (**H**) (engrailed) -**ii** Number of participating AB nuclei (dashed line) and PB nuclei (straight line) in Stripe 3, 4, and 5.

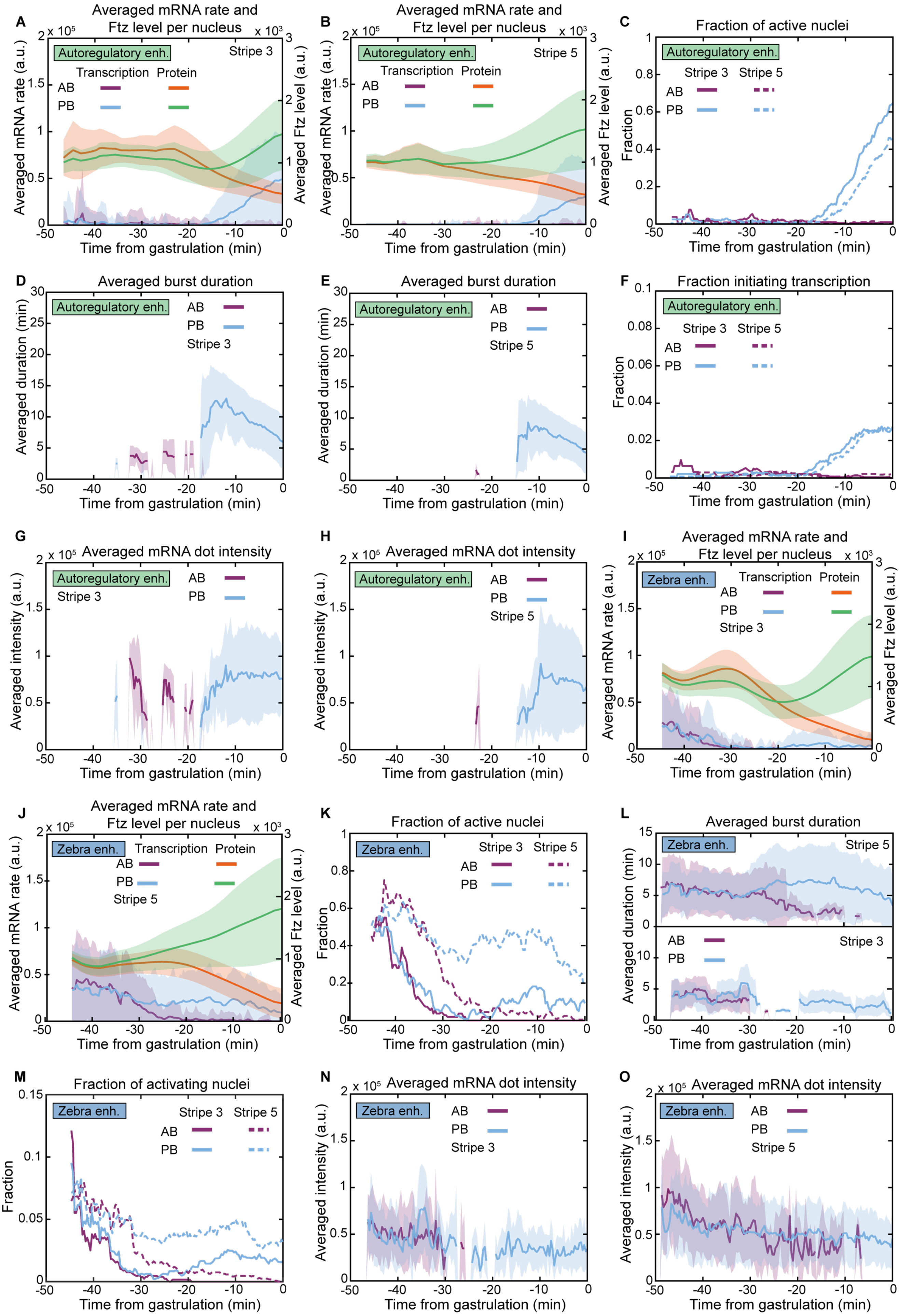
A
Averaged mRNA rate and Ftz level per nucleus
Autoregulatory enh.
Stripe 3
Transcription Protein
AB
PB
Averaged mRNA rate (a.u.)
Averaged Ftz level (a.u.)
Time from gastrulation (min)
B
Averaged mRNA rate and Ftz level per nucleus
Autoregulatory enh.
Stripe 5
C
Fraction of active nuclei
Autoregulatory enh.
Stripe 3 Stripe 5
Fraction
D
Averaged burst duration
Autoregulatory enh.
Stripe 3
Averaged duration (min)
E
Averaged burst duration
Autoregulatory enh.
Stripe 5
F
Fraction initiating transcription
Autoregulatory enh.
Stripe 3 Stripe 5
G
Averaged mRNA dot intensity
Autoregulatory enh.
Stripe 3
Averaged intensity (a.u.)
H
Averaged mRNA dot intensity
Autoregulatory enh.
Stripe 5
I
Averaged mRNA rate and Ftz level per nucleus
Zebra enh.
Stripe 3
J
Averaged mRNA rate and Ftz level per nucleus
Zebra enh.
Stripe 5
K
Fraction of active nuclei
Zebra enh.
L
Averaged burst duration
Zebra enh.
Stripe 5
Stripe 3
M
Fraction of activating nuclei
Zebra enh.
N
Averaged mRNA dot intensity
Zebra enh.
Stripe 3
O
Averaged mRNA dot intensity
Zebra enh.
Stripe 5

Figure S4. Transcription driven by the zebra and autoregulatory enhancer, stripe 3 and 5, Related to Figure 3.

(**A**) (Autoregulatory, Stripe 3), (**B**) (Autoregulatory, Stripe 5), (**I**) (Zebra, Stripe 3), (**J**) (Zebra, Stripe 5) Averaged mRNA rate per nucleus in AB and PB nuclei (left axis) together with the averaged Ftz levels in those nuclei (right axis). (**C**) (Autoregulatory), (**K**) (Zebra) The transcribing fraction of AB and PB nuclei in Stripe 3 (straight line) and Stripe 5 (dashed line). (**D**) (Autoregulatory, Stripe 3), (**E**) (Autoregulatory, Stripe 5), (**L**) (Zebra, Stripe 3 and 5) The averaged burst duration in AB and PB nuclei displaying transcription. (**F**) (Autoregulatory), (**M**) (Zebra)The fraction of AB and PB nuclei starting a transcription burst in Stripe 3 (straight line) and Stripe 5 (dashed line). (**G**) (Autoregulatory, Stripe 3), (**H**) (Autoregulatory, Stripe 5), (**N**) (Zebra, Stripe 3), (**O**) (Zebra, Stripe 5) The averaged intensity of mRNA dots in AB and PB nuclei displaying transcription. In panels A, B, D, E, G, H, I, J, L, N, and O thick lines indicate the mean over all included nuclei, while shading is the standard deviation. In panels C, F, K, and M the fraction is calculated over all included nuclei.

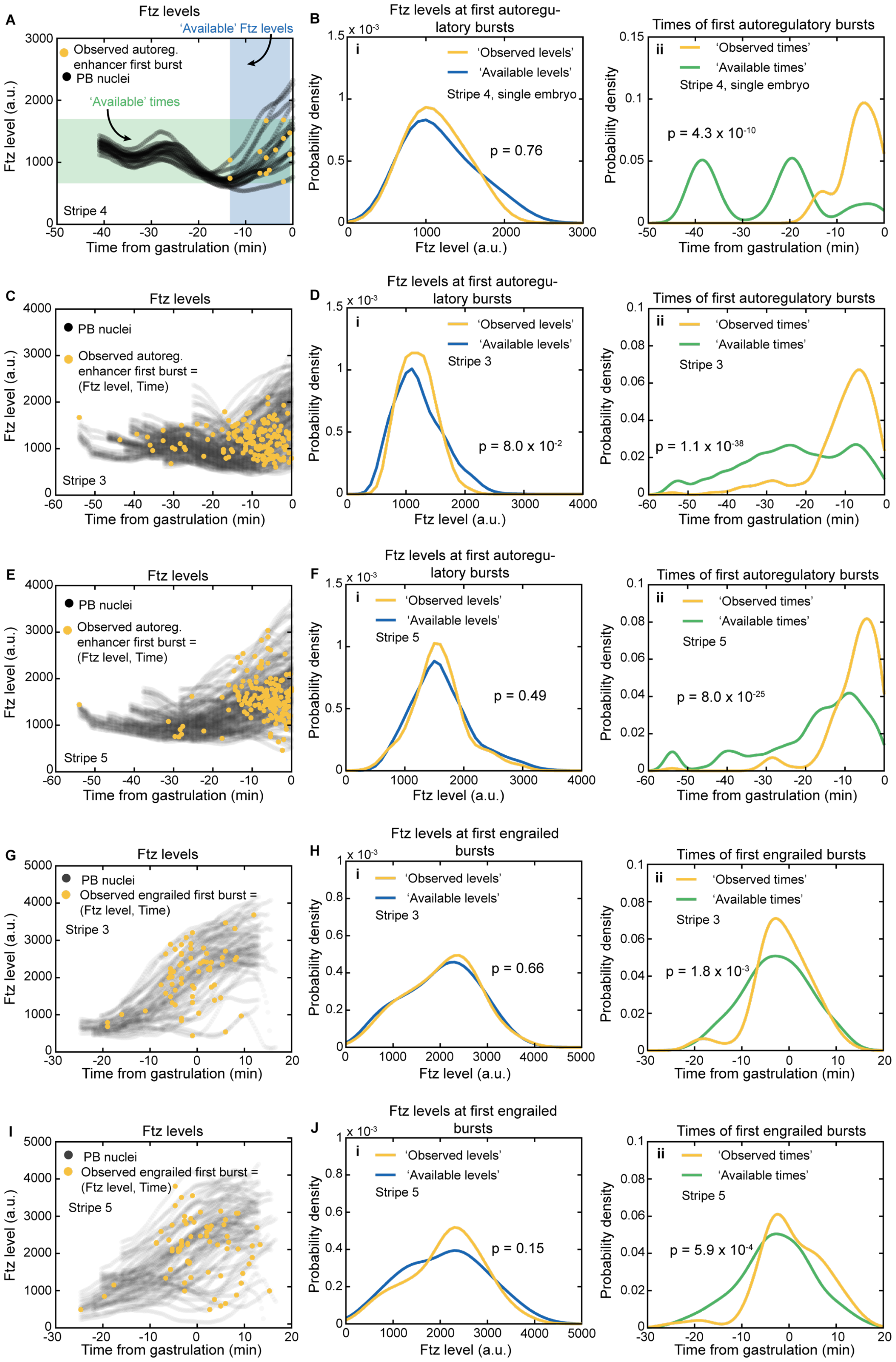
A
Ftz levels
'Available' Ftz levels
Observed autoreg. enhancer first burst
PB nuclei
'Available' times
Stripe 4
Ftz level (a.u.)
Time from gastrulation (min)
B
Ftz levels at first autoregulatory bursts
i
'Observed levels'
'Available levels'
Stripe 4, single embryo
p = 0.76
Probability density
Ftz level (a.u.)
Times of first autoregulatory bursts
ii
'Observed times'
'Available times'
Stripe 4, single embryo
p = 4.3 x 10^-10
Time from gastrulation (min)
C
Ftz levels
PB nuclei
Observed autoreg. enhancer first burst = (Ftz level, Time)
Stripe 3
D
Ftz levels at first autoregulatory bursts
i
'Observed levels'
'Available levels'
Stripe 3
p = 8.0 x 10^-2
Times of first autoregulatory bursts
ii
'Observed times'
'Available times'
Stripe 3
p = 1.1 x 10^-38
E
Ftz levels
PB nuclei
Observed autoreg. enhancer first burst = (Ftz level, Time)
Stripe 5
F
Ftz levels at first autoregulatory bursts
i
'Observed levels'
'Available levels'
Stripe 5
p = 0.49
Times of first autoregulatory bursts
ii
'Observed times'
'Available times'
Stripe 5
p = 8.0 x 10^-25
G
Ftz levels
PB nuclei
Observed engrailed first burst = (Ftz level, Time)
Stripe 3
H
Ftz levels at first engrailed bursts
i
'Observed levels'
'Available levels'
Stripe 3
p = 0.66
Times of first engrailed bursts
ii
'Observed times'
'Available times'
Stripe 3
p = 1.8 x 10^-3
I
Ftz levels
PB nuclei
Observed engrailed first burst = (Ftz level, Time)
Stripe 5
J
Ftz levels at first engrailed bursts
i
'Observed levels'
'Available levels'
Stripe 5
p = 0.15
Times of first engrailed bursts
ii
'Observed times'
'Available times'
Stripe 5
p = 5.9 x 10^-4

Figure S5. The Ftz levels and times at which the first mRNA burst in autoregulatory enhancer and engrailed embryos takes place, Related to Figure 4, 6 and STAR-methods.

(**A**) (stripe 4, single embryo, autoregulatory enhancer), (**C**) (stripe 3, autoregulatory enhancer), (**E**) (stripe 5, autoregulatory enhancer), (**G**) (stripe 3, engrailed), and (**I**) (stripe 5, engrailed) Scatter plot of Ftz levels (black), for all PB nuclei showing either autoregulatory enhancer or engrailed transcription. The Ftz level in each PB nucleus at the start of the first mRNA burst is annotated in yellow. (**B**) (stripe 4, single embryo, autoregulatory enhancer), (**D**) (stripe 3, autoregulatory enhancer), (**F**) (stripe 5, autoregulatory enhancer), (**H**) (stripe 3, engrailed) and (**J**) (stripe 5, engrailed) **-i** The observed Ftz levels (yellow) at the start of the first mRNA burst and the potentially available Ftz levels (blue) **-ii** The starting times (yellow) of the first mRNA burst and the potentially available timepoints (green). Two-sample Kolmogorov-Smirnov test was used for panels B, D, F, H, and J. Null-hypothesis = observed and 'available' values come from same distribution. Alternative hypothesis = 'available' values tend to be smaller than observed values. For panels B, D, F, H, J **-i**, the test at 5% significance does not reject the null hypothesis. For panel B, D, F, H, J -**ii**, the test does reject the null-hypothesis and accepts the alternative hypothesis.

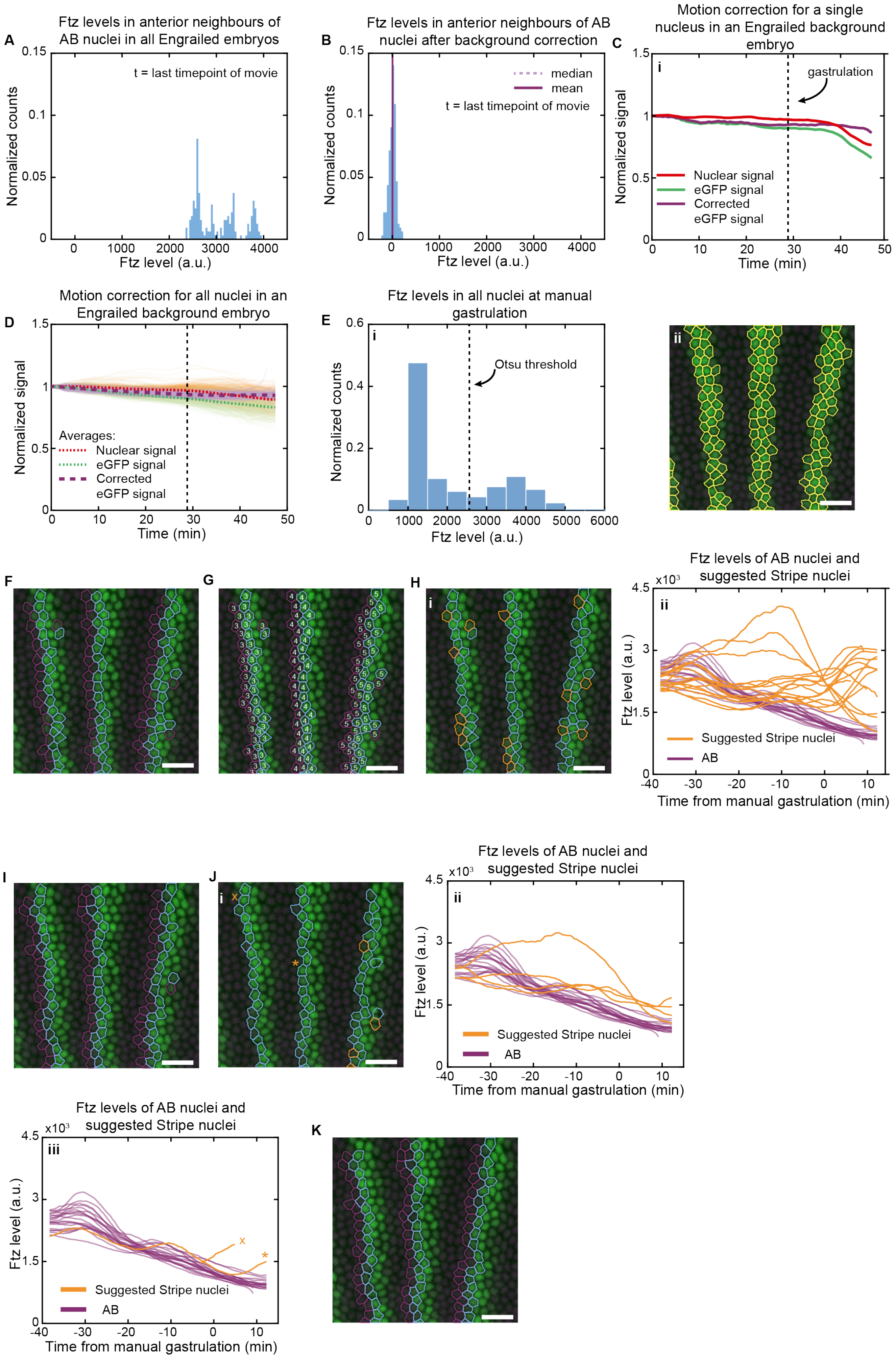

A
Ftz levels in anterior neighbours of AB nuclei in all Engrailed embryos
t = last timepoint of movie
Normalized counts
Ftz level (a.u.)
B
Ftz levels in anterior neighbours of AB nuclei after background correction
median
mean
t = last timepoint of movie
Normalized counts
Ftz level (a.u.)
C
Motion correction for a single nucleus in an Engrailed background embryo
i
gastrulation
Nuclear signal
eGFP signal
Corrected eGFP signal
Normalized signal
Time (min)
D
Motion correction for all nuclei in an Engrailed background embryo
Averages:
Nuclear signal
eGFP signal
Corrected eGFP signal
Normalized signal
Time (min)
E
Ftz levels in all nuclei at manual gastrulation
i
Otsu threshold
Normalized counts
Ftz level (a.u.)
ii
F
G
H
i
Ftz levels of AB nuclei and suggested Stripe nuclei
ii
Suggested Stripe nuclei
AB
Ftz level (a.u.)
Time from manual gastrulation (min)
I
J
i
Ftz levels of AB nuclei and suggested Stripe nuclei
ii
Suggested Stripe nuclei
AB
Ftz level (a.u.)
Time from manual gastrulation (min)
Ftz levels of AB nuclei and suggested Stripe nuclei
iii
Suggested Stripe nuclei
AB
Ftz level (a.u.)
Time from manual gastrulation (min)
K

Figure S6. Background subtraction and nuclear movement correction for engrailed embryos, and the algorithm to determine the Anterior and Posterior Boundary (AB and PB) nuclei, Related to Figure 1, 6, and STAR-methods.

(**A**) Histogram of the Ftz levels in the anteriorly positioned neighbours of AB nuclei at the final timepoint of each video, for all engrailed embryos. For each embryo, the mean of the embryo in question is used as its background value. (**B**) Histogram of the Ftz levels, after application of the background correction, in the anteriorly positioned neighbours of AB nuclei at the last timepoint of each video, for all engrailed embryos. The mean and median of the resulting distribution is centered around zero. (**C**) and (**D**) Background eGFP (green) and nuclear marker signal (red) for an example nucleus (**C**) and all nuclei (**D**) in an engrailed background embryo, normalized to their respective values at the first timepoint. By using the nuclear marker signal to normalize the background eGFP signal, the purple trace is obtained, which is corrected for nuclear movement and partially for bleaching. The means of the single nucleus traces for eGFP, nuclear marker and corrected eGFP signal are annotated using dashed green, red, and purple lines, respectively. The gastrulation time is annotated with a dashed black line. (**E**-**i**) Histogram of Ftz levels of all visible nuclei at manual gastrulation time, included the threshold on basis of which the nuclei are sorted in ‘stripe’ and ‘non-stripe’ nuclei. -**ii** The initial guess of ‘stripe’ nuclei. (**F**) The result of the first round of AB (Purple) and PB (Blue) nuclei classification. (**G**) Stripe numbering of the initial guess of AB and PB nuclei. (**H**) First round of refinement. -**i** List of suggested nuclei (Orange), which might be ‘stripe’ nuclei instead of ‘non-stripe’ nuclei. -**ii** Ftz level traces of these suggested nuclei, together with traces of AB nuclei. (**I**) The second guess of AB and PB nuclei. (**J**) Second round of refinement. -**i** List of suggested nuclei, which might be ‘stripe’ instead of ‘non-stripe’ nuclei. Also, annotated are two nuclei (x and *) which were missed by the automated suggestions. -**ii** Ftz level traces of the automatically suggested nuclei, together with traces of AB nuclei. -**iii** Ftz level traces of the manually suggested nuclei (corresponding to x and * in **J**-**i**), together with traces of AB nuclei. (**K**) The final classification of the AB and PB nuclei. For all images: green channel is Ftz and red channel is MCP-mCherry nuclear signal. Scale bars are 20 μm.

Supplemental References

S1 MacArthur, S. *et al.* Developmental roles of 21 Drosophila transcription factors are determined by quantitative differences in binding to an overlapping set of thousands of genomic regions. *Genome Biol.* **10**, R80 (2009).

S2 Harrison, M. M., Li, X.-Y., Kaplan, T., Botchan, M. R. & Eisen, M. B. Zelda Binding in the Early Drosophila melanogaster Embryo Marks Regions Subsequently Activated at the Maternal-to-Zygotic Transition. *PLoS Genet.* **7**, e1002266 (2011).

S3 Soluri, I. V., Zumerling, L. M., Parra, O. A. P., Clark, E. G. & Blythe, S. A. Zygotic pioneer factor activity of odd-paired/zic is necessary for late function of the drosophila segmentation network. *Elife* **9**, 1–36 (2020).
